# On the Quasistatic Limit of Dynamic Evolutions for a Peeling Test in Dimension One

**DOI:** 10.1007/s00332-017-9407-0

**Published:** 2017-08-17

**Authors:** Giuliano Lazzaroni, Lorenzo Nardini

**Affiliations:** 1 Faculty of Mathematics, University of Vienna, Oskar-Morgenstern-Platz 1, 1090 Vienna, Austria; 2 SISSA, via Bonomea 265, 34136 Trieste, Italy

**Keywords:** Griffith’s criterion, Dynamic debonding, Quasistatic limit, Rate-independent evolution, Singular perturbation, Vanishing inertia, 35L05, 35B40, 35Q74, 35R35, 74J40, 74K35

## Abstract

The aim of this paper is to study the quasistatic limit of a one-dimensional model of dynamic debonding. We start from a dynamic problem that strongly couples the wave equation in a time-dependent domain with Griffith’s criterion for the evolution of the domain. Passing to the limit as inertia tends to zero, we find that the limit evolution satisfies a stability condition; however, the activation rule in Griffith’s (quasistatic) criterion does not hold in general, thus the limit evolution is not rate-independent.

## Introduction

In models that predict the growth of cracks in structures, it is often assumed that the process is quasistatic. The quasistatic hypothesis is that inertial effects can be neglected since the time scale of the external loading is very slow, or equivalently the speed of the internal oscillations is very large if compared with the speed of loading. The resulting evolutions are rate-independent, i.e. the system is invariant under time reparametrisation.

Starting from the scheme proposed in Francfort and Marigo (1998), quasistatic crack growth has been extensively studied in the mathematical literature. The existence of quasistatic evolutions in fracture mechanics has been proved in several papers concerning globally minimising evolutions (Dal Maso and Toader 2002; Chambolle 2003; Francfort and Larsen 2003; Dal Maso et al. 2005; Dal Maso and Zanini 2007; Dal Maso and Lazzaroni 2010; Cagnetti and Toader 2011; Lazzaroni 2011; Crismale et al. 2016) and vanishing-viscosity solutions (Negri and Ortner 2008; Cagnetti 2008; Knees et al. 2008, 2010; Lazzaroni and Toader 2011; Artina et al. 2017; Almi 2017; Crismale and Lazzaroni 2016). We refer to Bourdin et al. (2008) for a presentation of the variational approach to fracture and to Mielke and Roubíček (2015) for the relations with the abstract theory of rate-independent systems. These results also show that quasistatic evolutions may present phases of brutal crack growth (appearing as time discontinuities in the quasistatic scale). In order to study fast propagations of cracks, a dynamical analysis is needed, since inertial effects have to be accounted for.

On the other hand, in the case of dynamic fracture, only preliminary existence results were given (Nicaise and Sändig 2007; Dal Maso and Larsen 2011; Dal Maso and Lucardesi 2016; Dal Maso et al. 2016). The main difficulty is that the equations of elastodynamics for the displacement have to be satisfied in a time-dependent domain (i.e. the body in its reference configuration, minus the growing crack), while the evolution of the domain is prescribed by a first-order flow rule. The resulting PDE system is strongly coupled, as in other models of damage or delamination [see, e.g. Frémond and Nedjar (1996), Bonetti et al. (2005), Bonetti and Bonfanti (2008), Rocca and Rossi (2014, 2015), Heinemann and Kraus (2015b, 2015a) for viscous flow rules and Roubíček (2009, 2010, 2013a, b), Larsen et al. (2010), Rossi and Roubíček (2011), Bartels and Roubíček (2011), Babadjan and Mora (2015), Lazzaroni et al. (2014), Roubíček and Tomassetti (2015), Maggiani and Mora (2016) and Rossi and Thomas (2016) for rate-independent evolutions of internal variables].

In few cases, it has been shown that the quasistatic hypothesis is a good approximation, that is, the dynamic solutions converge to a rate-independent evolution as inertia tends to zero. This was proved in Roubíček (2013a) and Lazzaroni et al. (2014) for damage models, including a damping term in the wave equation, and in Dal Maso and Scala (2014) in the case of perfect plasticity. On the other hand, even in finite dimension there are examples of singularly perturbed second-order potential-type equations (where the inertial term vanishes and the formal limit is an equilibrium equation), such that the dynamic solutions do not converge to equilibria (Nardini 2017). In finite dimension, if the equations include a friction term whose coefficient tends to zero as inertia vanishes, then the dynamic evolutions converge to a solution of the equilibrium equation (Agostiniani 2012).

In this paper we develop a “vanishing inertia” analysis for a model of dynamic debonding in dimension one. More precisely, we consider a peeling test for a perfectly flexible thin film initially attached to a rigid substrate; the process is assumed to depend only on one of the two variables parametrising the film. This model was studied in Dumouchel et al. (2008), Lazzaroni et al. (2012) and Dal Maso et al. (2016) and, as already observed in Freund (1990, Section 7.4), it is related to dynamic fracture since it features a coupling between the wave equation, satisfied in the debonded part of the film, and a flow rule for the evolution of the debonding front.

**Fig. 1:**
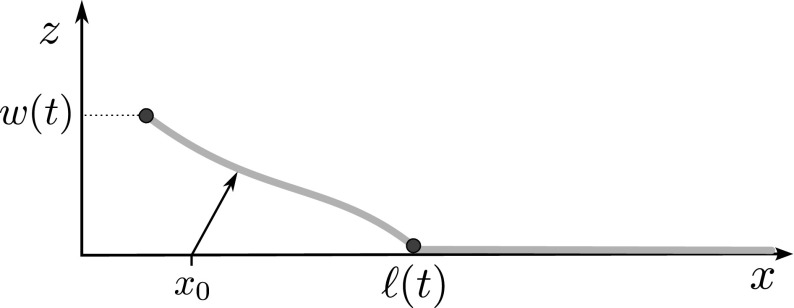
The *curve* $x \mapsto (x+h(t,x), u(t,x))$ representing the debonding of the film. The displacement associated to the point $x_0$ is $(h(t,x_0),u(t,x_0))$

We now describe the mechanical system under consideration and the related mathematical problem. In an orthogonal coordinate system (*x*, *y*, *z*), the film is parametrised on the half plane $\{(x,y,z) : x\ge 0,\, z=0\}$. Its deformation at time $t\ge 0$ is given by $(x,y,0) \mapsto (x{+}h(t,x), y, u(t,x))$. Specifically, the deformed configuration is parametrised by the scalar functions *h* and *u*, while the second component is assumed to be constant and therefore it will be ignored in the following discussion. See Fig. 1.

The film is partially bonded to the rigid substrate $\{(x,0) : x\ge 0\}$. In the reference configuration the debonded region is $\{(x,0) : x \le \ell (t)\}$, where $t\mapsto \ell (t)$ is a non-decreasing function satisfying the initial condition $\ell _0:=\ell (0)>0$. As a consequence, for $x \ge \ell (t)$ we have $h(t,x)=u(t,x)=0$. At the endpoint $x=0$ the vertical displacement *u*(*t*, 0) is prescribed. Assuming inextensibility, by linear approximation we obtain

$$\begin{aligned} h(t,x) = \frac{1}{2} \int _x^{+\infty } u_x(t,\xi )^2\mathrm {d}\xi . \end{aligned}$$

Then the unknowns of the problem are the vertical displacement *u* and the debonding front $\ell$.

In this work we study the behaviour of this system when the speed of loading and the initial velocity of the displacement are very small. More precisely, the prescribed vertical displacement is given by $w_\varepsilon (t):=w(\varepsilon t)$ where *w* is a given function and $\varepsilon >0$ is a small parameter. The initial vertical displacement and its initial velocity are, respectively, $u_0$ and $\varepsilon u_1$, where $u_0$ and $u_1$ are two functions of *x* satisfying some suitable assumptions. We use the notation $(u_\varepsilon ,\ell _\varepsilon )$ to underline the dependence of the solution on $\varepsilon$. Assuming that the speed of sound is constant and normalised to one, the problem satisfied by $u_\varepsilon$ is

1.1a $$\begin{aligned}&(u_\varepsilon )_{tt}(t,x)-(u_\varepsilon )_{xx}(t,x)=0,&t > 0,\; 0<x<\ell _\varepsilon (t), \end{aligned}$$

1.1b $$\begin{aligned}&u_\varepsilon (t,0)=w_\varepsilon (t),&t>0, \end{aligned}$$

1.1c $$\begin{aligned}&u_\varepsilon (t,\ell _\varepsilon (t))=0,&t>0,\end{aligned}$$

1.1d $$\begin{aligned}&u_\varepsilon (0,x)=u_0(x),&0<x<\ell _0, \end{aligned}$$

1.1e $$\begin{aligned}&(u_\varepsilon )_t(0,x)=\varepsilon u_1(x),&0<x<\ell _0. \end{aligned}$$

In the paper we consider a more general dependence of *w*, $u_0$, and $u_1$ on $\varepsilon$, see (2.1).

The evolution of the debonding front $\ell _\varepsilon$ is determined by a criterion involving the internal energy, i.e. the sum of the potential and the kinetic energy, cf. (2.8). Specifically, this criterion involves the dynamic energy release rate, which is defined as a (sort of) partial derivative of the internal energy with respect to the elongation of the debonded region. We refer to the following section for its definition and for details on its existence, which was proved in Dal Maso et al. (2016). In this introduction we only stress that the dynamic energy release rate at time *t*, denoted by $G_\varepsilon (t)$, depends only on the debonding speed $\dot{\ell }_\varepsilon (t)$ and on the values of $u_\varepsilon (s,x)$ for $s\le t$.

The flow rule for the evolution of the debonding front is called Griffith’s criterion and reads as

1.2a $$\begin{aligned}&\dot{\ell }_\varepsilon (t)\ge 0,\end{aligned}$$

1.2b $$\begin{aligned}&G_\varepsilon (t)\le \kappa (\ell _\varepsilon (t)),\end{aligned}$$

1.2c $$\begin{aligned}&\dot{\ell }_\varepsilon (t)\left[ G_\varepsilon (t)-\kappa (\ell _\varepsilon (t))\right] =0, \end{aligned}$$

for a.e. $t>0$, where $\kappa :[0,+\infty )\rightarrow [c_1,c_2]$, $0<c_1<c_2$, is the local toughness of the glue between the film and the substrate and $\ell _\varepsilon (0)=\ell _0$. This criterion is a consequence of a maximum dissipation principle, see also Dal Maso et al. (2016, Section 2.2), and is a condition of Kuhn–Tucker type. Indeed, Eq. (1.2c) states that (1.2a) may hold as a strict inequality only if (1.2b) holds as an equality.

The existence of a unique solution $(u_\varepsilon , \ell _\varepsilon )$ in a weak sense was proved under suitable assumptions in Dal Maso et al. (2016), see also below. Notice the strong coupling between (1.1) and (1.2): indeed, the variable $\ell _\varepsilon$ appears in the domain of (1.1a), while $G_\varepsilon$ in (1.2) depends on $u_\varepsilon$. This is typical of dynamic fracture, too.

In fact, the peeling test is closely related to fracture. The debonded part of the film, here parametrised on the interval $(0,\ell _\varepsilon (t))$, corresponds to the uncracked part of a body subject to fracture; both domains are monotone in time, though in opposite directions, increasing in our case, decreasing in the fracture problem. The debonding propagation $t\mapsto \ell _\varepsilon (t)$ corresponds to the evolution of a crack tip. The debonding front $\ell _\varepsilon (t)$ has the role of a free boundary just as a crack. However, notice that cracks are discontinuity sets for the displacement, where a homogeneous Neumann condition is satisfied since they are traction free; in contrast, in the peeling test the displacement is continuous at $\ell _\varepsilon (t)$ because of the Dirichlet constraint (1.1c): the debonding front is a discontinuity set for the displacement derivatives and represents a free boundary between $\{x: u_\varepsilon (x,s)=0 \text { for every } s\le t \}$ and $\{x: u_\varepsilon (x,s)\ne 0 \text { for some } s\le t\}$.

In this work we perform an asymptotic analysis of (1.1) and (1.2) as $\varepsilon$ tends to zero, i.e. we study the limit of the system for quasistatic loading. Some results in this direction were given in Dumouchel et al. (2008) and Lazzaroni et al. (2012) in the specific case of a piecewise constant toughness.

It is convenient to consider the rescaled functions

$$\begin{aligned} (u^\varepsilon (t,x),\ell ^\varepsilon (t)):=\left( u_\varepsilon \left( \frac{t}{\varepsilon },x\right) ,\ell _\varepsilon \left( \frac{t}{\varepsilon }\right) \right) . \end{aligned}$$

After this time rescaling, the problem solved by $(u^\varepsilon ,\ell ^\varepsilon )$ consists of the equation of elastodynamics complemented with initial and boundary conditions

1.3a $$\begin{aligned}&\varepsilon ^2u^\varepsilon _{tt}(t,x)-u^\varepsilon _{xx}(t,x)=0,&t>0,\; 0<x<\ell ^\varepsilon (t), \end{aligned}$$

1.3b $$\begin{aligned}&u^\varepsilon (t,0)=w(t),&t>0, \end{aligned}$$

1.3c $$\begin{aligned}&u^\varepsilon (t,\ell ^\varepsilon (t))=0,&t>0,\end{aligned}$$

1.3d $$\begin{aligned}&u^\varepsilon (0,x)=u_0(x),&0<x<\ell _0, \end{aligned}$$

1.3e $$\begin{aligned}&u^\varepsilon _t(0,x)= u_1(x),&0<x<\ell _0, \end{aligned}$$

and coupled with Griffith’s criterion

1.4a $$\begin{aligned}&\dot{\ell }^\varepsilon (t)\ge 0,\end{aligned}$$

1.4b $$\begin{aligned}&G^\varepsilon (t)\le \kappa (\ell ^\varepsilon (t)),\end{aligned}$$

1.4c $$\begin{aligned}&\dot{\ell }^\varepsilon (t)\left[ G^\varepsilon (t)-\kappa (\ell ^\varepsilon (t))\right] =0, \end{aligned}$$

where $G^\varepsilon (t) = G_\varepsilon (\frac{t}{\varepsilon })$ and $\ell ^\varepsilon (0)=\ell _0$. Notice that the speed of sound is now $\frac{1}{\varepsilon }$. Indeed, in the quasistatic limit the timescale of the internal oscillations is much faster than the timescale of the loading.

The existence of a unique solution $(u^\varepsilon ,\ell ^\varepsilon )$ to the coupled problem (1.3) and (1.4) for a fixed $\varepsilon >0$ is guaranteed by Dal Maso et al. (2016, Theorem 3.5), provided the data are Lipschitz and the local toughness is piecewise Lipschitz; moreover it turns out that $u^\varepsilon$ is Lipschitz in time and space and $\ell ^\varepsilon$ is Lipschitz in time. (See also Theorem 2.3 below.) The strategy employed there to prove the existence result relies on the specific one-dimensional setting of the model. Indeed, it is possible to write the solution $u^\varepsilon$ of the wave equation (1.3a) in terms of a one-dimensional function $f^\varepsilon$; more precisely, $u^\varepsilon (t,x)$ depends on $f^\varepsilon (x\pm \varepsilon t)$ through the D’Alembert formula (2.4). On the other hand, the dynamic energy release rate $G^\varepsilon$ can also be expressed as a function of $f^\varepsilon$, so Griffith’s criterion (1.4) reduces to a Cauchy problem which has a unique solution.

In this work, in order to study the limit of the solutions $(u^\varepsilon ,\ell ^\varepsilon )$ as $\varepsilon \rightarrow 0$, we use again the one-dimensional structure of the model. First we derive an a priori bound for the internal energy, uniform with respect to $\varepsilon$; to this end, it is convenient to write the internal energy in terms of $f^\varepsilon$, see Proposition 3.1. The uniform bound allows us to find a limit pair $(u,\ell )$. More precisely, since the functions $\ell ^\varepsilon$ are non-decreasing and $\ell ^\varepsilon (T)<L$, Helly’s Theorem provides a subsequence $\varepsilon _k$ such that $\ell ^{\varepsilon _k}$ converges for every *t* to a (possibly discontinuous) non-decreasing function $\ell$. On the other hand, the uniform bound on $u^{\varepsilon _k}$ in $L^2(0,T;H^1(0,L))$ guarantees the existence of a weak limit *u*. We call $(u,\ell )$ the quasistatic limit of $(u^\varepsilon ,\ell ^\varepsilon )$.

The issue is now to pass to the limit in (1.3) and (1.4) and to understand the properties of the quasistatic limit. As for the vertical displacement, in our first main result (Theorem 3.5) we find that the equilibrium equations are satisfied, i.e. *u* solves the limit problem

1.5a $$\begin{aligned}&u_{xx}(t,x)=0,&t>0,\; 0<x<\ell (t), \end{aligned}$$

1.5b $$\begin{aligned}&u(t,0)=w(t),&t>0, \end{aligned}$$

1.5c $$\begin{aligned}&u(t,\ell (t))=0,&t>0. \end{aligned}$$

More precisely, for a.e. *t*, $u(t,\cdot )$ is affine in $(0,\ell (t))$ and $u(t,x)=-\frac{w(t)}{\ell (t)}x+w(t)$. To prove this, we exploit a technical lemma stating that the graphs of $\ell ^{\varepsilon _k}$ converge to the graph of $\ell$ in the Hausdorff metric, see Lemma 3.4. We remark that in general the initial conditions (1.3d) and (1.3e) do not pass to the limit since there may be time discontinuities, even at $t=0$.

Next we study the flow rule satisfied by the limit debonding evolution $\ell$. We question whether it complies with the quasistatic formulation of Griffith’s criterion,

1.6a $$\begin{aligned}&\dot{\ell }(t)\ge 0,\end{aligned}$$

1.6b $$\begin{aligned}&G_\mathrm{qs}(t)\le \kappa (\ell (t)),\end{aligned}$$

1.6c $$\begin{aligned}&\left[ G_\mathrm{qs}(t)-\kappa (\ell (t))\right] \dot{\ell }(t)=0, \end{aligned}$$

where $G_\mathrm{qs}$ is the quasistatic energy release rate, that is, the partial derivative of the quasistatic internal energy with respect to the elongation of the debonded region. Notice that in the quasistatic setting the internal energy consists of the potential term only, so (1.6) is the formal limit of (1.4) as $\varepsilon \rightarrow 0$.

Condition (1.6a) is guaranteed by Helly’s Theorem. By passing to the limit in (1.4b), we also prove that (1.6b) holds. For this result we use again the D’Alembert formula for $u^\varepsilon$ and find the limit *f* of the one-dimensional functions $f^\varepsilon$. In fact, $\dot{f}$ turns out to be related to $u_x$ through an explicit formula, as we see in Theorem 3.11, which is our second main result.

In contrast, (1.6c) is in general not satisfied. This was already observed in the earlier paper (Lazzaroni et al. 2012), which presents an example of dynamic solutions whose limit violates (1.6c). The singular behaviour of these solutions is due to the choice of a toughness with discontinuities. Indeed, when the debonding front meets a discontinuity in the toughness, a shock wave is generated. The interaction of such singularities causes strong high-frequency oscillations of the kinetic energy, which affects the limit as the wave speed tends to infinity.

In the present paper, we continue the discussion of this kind of behaviour by providing an explicit case where (1.6c) does not hold in the limit even if the local toughness is constant and the other data are smooth. (See Sect. 4 and Remark 4.2). In our new example, the initial conditions are not at equilibrium, in particular the initial position $u_0$ is not affine in $(0,\ell _0)$. Therefore, due to the previous results, the quasistatic limit cannot satisfy the initial condition, i.e. it has a time discontinuity at $t=0$. Moreover, our analysis of the limit evolution $(u,\ell )$ shows that the internal energy given through the initial conditions is not relaxed instantaneously; its effects persist in a time interval where the evolution does not satisfy (1.6c). The surplus of initial energy, instantaneously converted into kinetic energy, cannot be quantified in a purely quasistatic analysis. For this reason the usual quasistatic formulation (1.6) is not suited to describe the quasistatic limit of our dynamic process.

## Existence and Uniqueness Results

In this section we provide an outline of the results of existence and uniqueness for the coupled problem (1.3) and (1.4) for fixed $\varepsilon >0$, proved in Dal Maso et al. (2016). The only difference with respect to Dal Maso et al. (2016) is that the speed of sound is $\frac{1}{\varepsilon }$ instead of 1.

We consider the following generalisation of problem (1.3),

2.1a $$\begin{aligned}&\varepsilon ^2u^\varepsilon _{tt}(t,x)-u^\varepsilon _{xx}(t,x)=0,&t > 0,\; 0<x<\ell ^\varepsilon (t), \end{aligned}$$

2.1b $$\begin{aligned}&u^\varepsilon (t,0)=w^\varepsilon (t),&t>0, \end{aligned}$$

2.1c $$\begin{aligned}&u^\varepsilon (t,\ell ^\varepsilon (t))=0,&t>0\end{aligned}$$

2.1d $$\begin{aligned}&u^\varepsilon (0,x)=u_0^\varepsilon (x),&0<x<\ell _0, \end{aligned}$$

2.1e $$\begin{aligned}&u^\varepsilon _t(0,x)= u_1^\varepsilon (x),&0<x<\ell _0. \end{aligned}$$

We require that

2.2a $$\begin{aligned} w^\varepsilon \in \widetilde{C}^{0,1}(0,+\infty ), \quad u_0^\varepsilon \in C^{0,1}([0,\ell _0]), \quad u_1^\varepsilon \in L^\infty (0,\ell _0), \end{aligned}$$

where

$$\begin{aligned} \widetilde{C}^{0,1} (0,+\infty ) := \{f \in C^0([0,+\infty )) : f \in C^{0,1}([0,T]) \text { for every } T>0\}, \end{aligned}$$

and the compatibility conditions

2.2b $$\begin{aligned} u_0^\varepsilon (0)=w^\varepsilon (0), \quad u_0^\varepsilon (\ell _0)=0. \end{aligned}$$

To give the notion of solution, for the moment we assume that the evolution of the debonding front $t\mapsto \ell ^\varepsilon (t)$ is known. More precisely, we fix $\ell _0>0$ and $\ell ^\varepsilon :[0,+\infty ) \rightarrow [\ell _0,+\infty )$ Lipschitz and such that

2.3a $$\begin{aligned}&0\le \dot{\ell }^\varepsilon (t)<\frac{1}{\varepsilon }, \quad \text{ for } \text{ a.e. } t>0, \end{aligned}$$

2.3b $$\begin{aligned}&\ell ^\varepsilon (0)=\ell _0. \end{aligned}$$

We introduce the sets

$$\begin{aligned} \Omega ^\varepsilon&:= \{(t,x) :t>0, \quad 0<x<\ell ^\varepsilon (t)\},\\ \Omega ^\varepsilon _T&:= \{(t,x) :0<t<T, \quad 0<x<\ell ^\varepsilon (t)\} \end{aligned}$$

and the spaces

$$\begin{aligned} \widetilde{H}{^1}(\Omega ^\varepsilon )&:= \{u \in H_{\hbox {loc}}^1(\Omega ^\varepsilon ): u \in H^1(\Omega ^\varepsilon _T), \text{ for } \text{ every } T>0\},\\ \widetilde{C}^{0,1}(\Omega ^\varepsilon )&:= \{u \in C^0(\Omega ^\varepsilon ): u \in C^{0,1}(\Omega ^\varepsilon _T) \text{ for } \text{ every } T>0\}, \end{aligned}$$

The notion of solution is given in the following sense.

### Definition 2.1

We say that $u^\varepsilon \in \widetilde{H}^1(\Omega ^\varepsilon )$ (resp. in $u^\varepsilon \in H^1(\Omega ^\varepsilon _T)$) is a solution to (2.1) if $\varepsilon ^2u^\varepsilon _{tt}-u^\varepsilon _{xx}=0$ holds in the sense of distributions in $\Omega ^\varepsilon$ (resp. $\Omega ^\varepsilon _T$), the boundary conditions (2.1b) and (2.1c) are intended in the sense of traces and the initial conditions (2.1d) and (2.1e) are satisfied in the sense of $L^2(0,\ell _0)$ and $H^{-1}(0,\ell _0)$, respectively.

Condition (2.1e) makes sense since $u_x^\varepsilon \in L^2(0,T;L^2(0,\ell _0))$ and, by the wave equation, $u_{xx}^\varepsilon , u_{tt}^\varepsilon \in L^2(0,T;H^{-1}(0,\ell _0))$, therefore $u_t^\varepsilon \in H^1(0,T;H^{-1}(0,\ell _0)) \subset C^0([0,T];H^{-1}(0,\ell _0))$. Arguing as in Dal Maso et al. (2016, Section 1), it is possible to uniquely solve (2.1) by means of the D’Alembert formula, as it is stated in the next proposition.

### Proposition 2.2

Assume (2.2) and (2.3). Then, there exists a unique solution $u^\varepsilon \in H^1(\Omega ^\varepsilon )$ to problem (2.1), according to Definition 2.1. Moreover, $u^\varepsilon \in \widetilde{C}^{0,1}(\Omega ^\varepsilon )$ and is expressed through the formula

2.4 $$\begin{aligned} u^\varepsilon (t,x)= w^\varepsilon (t{+}\varepsilon x) -\frac{1}{\varepsilon }f^\varepsilon (t{+}\varepsilon x)+\frac{1}{\varepsilon }f^\varepsilon (t{-}\varepsilon x), \end{aligned}$$

where $f^\varepsilon \in \widetilde{C}^{0,1}(-\varepsilon \ell _0,+\infty )$ is determined by

2.5 $$\begin{aligned} w^\varepsilon (t+\varepsilon \ell ^\varepsilon (t))-\frac{1}{\varepsilon }f^\varepsilon (t+\varepsilon \ell ^\varepsilon (t))+\frac{1}{\varepsilon }f^\varepsilon (t-\varepsilon \ell ^\varepsilon (t))=0, \quad \text{ for } \text{ every } t>0, \end{aligned}$$

and

2.6a $$\begin{aligned} f^\varepsilon (s) =&\,\varepsilon w^\varepsilon (s) -\frac{\varepsilon }{2}u_0^\varepsilon \left( \frac{s}{\varepsilon }\right) -\frac{\varepsilon ^2}{2}\int _0^{\frac{s}{\varepsilon }} u_1^\varepsilon (x) \mathrm {d}x - \varepsilon w^\varepsilon (0)+ \frac{\varepsilon }{2}u_0^\varepsilon (0),\nonumber \\&\quad \text{ for } \text{ every } s \in [0,\varepsilon \ell _0],\end{aligned}$$

2.6b $$\begin{aligned} f^\varepsilon (s) =&\,\frac{\varepsilon }{2}u_0^\varepsilon \left( -\frac{s}{\varepsilon }\right) - \frac{\varepsilon ^2}{2} \int _0^{-\frac{s}{\varepsilon }} u_1^\varepsilon (x) \mathrm {d}x -\frac{\varepsilon }{2}u_0^\varepsilon (0), \quad \text{ for } \text{ every } s \in (-\varepsilon \ell _0,0]. \end{aligned}$$

By derivation of (2.4) we obtain

2.7a $$\begin{aligned} u_t^\varepsilon (t,x)&= \dot{w}^\varepsilon (t{+}\varepsilon x) - \frac{1}{\varepsilon }\dot{f}^\varepsilon (t{+}\varepsilon x) + \frac{1}{\varepsilon }\dot{f}^\varepsilon (t{-}\varepsilon x), \end{aligned}$$

2.7b $$\begin{aligned} u_x^\varepsilon (t,x)&= \varepsilon \dot{w}^\varepsilon (t{+}\varepsilon x) - \dot{f}^\varepsilon (t{+}\varepsilon x) - \dot{f}^\varepsilon (t{-}\varepsilon x) . \end{aligned}$$

Formula (2.7a) guarantees that, for every *t*, $u^\varepsilon _t(t,\cdot )$ is defined a.e. in $(0,\ell ^\varepsilon (t))$. In this paper we always use $\dot{f}$ to indicate the derivative of a function *f* of only one variable (even if that variable is not the time).

The last observation and the existence of a unique solution to (2.1) stated in Proposition 2.2 allow us to define the internal energy

2.8 $$\begin{aligned} \mathcal {E}^\varepsilon (t;\ell ^\varepsilon ,w^\varepsilon ):= \int _0^{\ell ^\varepsilon (t)}\left[ \frac{\varepsilon ^2}{2} u^\varepsilon _t(t,x)^2 + \frac{1}{2} u^\varepsilon _x(t,x)^2 \right] \mathrm {d}x. \end{aligned}$$

In the previous expression the internal energy is a functional of $\ell ^\varepsilon$ and $w^\varepsilon$, while $u^\varepsilon$ is the unique solution of (2.1) corresponding to the prescribed debonding evolution $\ell ^\varepsilon$ and to the data of the problem. Using (2.7), then (2.8) reads as

2.9 $$\begin{aligned} \mathcal {E}^\varepsilon (t;\ell ^\varepsilon ,w^\varepsilon )=\frac{1}{\varepsilon }\int _{t}^{t+\varepsilon \ell ^\varepsilon (t)}[\varepsilon \dot{w}^\varepsilon (s) - \dot{f}^\varepsilon (s)]^2\mathrm {d}s + \frac{1}{\varepsilon }\int _{t-\varepsilon \ell ^\varepsilon (t)}^{t} \dot{f}^\varepsilon (s)^2\mathrm {d}s. \end{aligned}$$

We now give the notion of dynamic energy release rate which is used to give the criterion for the (henceforth unknown) evolution of the debonding front $\ell ^\varepsilon$. Specifically, the dynamic energy release rate $G^\varepsilon _\alpha (t_0)$ at time $t_0$ corresponding to a speed $0<\alpha <\frac{1}{\varepsilon }$ of the debonding front is defined as

$$\begin{aligned} G_\alpha ^\varepsilon (t_0) := \lim _{t\rightarrow t_0^+} \frac{\mathcal {E}^\varepsilon (t_0;\lambda ^\varepsilon ,z^\varepsilon ) - \mathcal {E}^\varepsilon (t;\lambda ^\varepsilon ,z^\varepsilon )}{(t-t_0)\alpha }, \end{aligned}$$

where $\lambda ^\varepsilon \in C^{0,1}([0,+\infty ))$ is such that $\lambda ^\varepsilon (t) = \ell ^\varepsilon (t)$ for every $0\le t \le t_0$, $\dot{\lambda }^\varepsilon <\frac{1}{\varepsilon }$ for a.e. $t>0$, and

$$\begin{aligned} \frac{1}{h} \int _{t_0}^{t_0+h} \left| \dot{\lambda ^\varepsilon }(t) - \alpha \right| \mathrm {d}t \rightarrow 0, \quad \text { as } h \rightarrow 0^+, \end{aligned}$$

while

$$\begin{aligned} z^\varepsilon (t)={\left\{ \begin{array}{ll} w^\varepsilon (t), &{}\; t\le t_0, \\ w^\varepsilon (t_0), &{} \;t>t_0. \end{array}\right. } \end{aligned}$$

In Dal Maso et al. (2016, Section 2), it is proved that, given $\ell ^\varepsilon$ and $w^\varepsilon$, the limit above exists for a.e. $t_0 >0$ and for every $\alpha \in (0,\frac{1}{\varepsilon })$. Moreover, it is expressed in terms of $f^\varepsilon$ through the following formula:

2.10 $$\begin{aligned} G^\varepsilon _{\alpha }(t) = 2\frac{1-\varepsilon \alpha }{1+\varepsilon \alpha }\dot{f}^\varepsilon (t {-}\varepsilon \ell ^\varepsilon (t))^2. \end{aligned}$$

This also shows that $G^\varepsilon _\alpha$ depends on the choice of $\lambda ^\varepsilon$ only through $\alpha$, and therefore, the definition is well posed. We also extend by continuity this definition to the case $\alpha =\dot{\ell }^\varepsilon (t)=0$, by setting

$$\begin{aligned} G^\varepsilon _0(t):=2\dot{f}^\varepsilon (t{-}\varepsilon \ell ^\varepsilon (t))^2. \end{aligned}$$

Thus, by (2.10), we have monotonicity with respect to $\alpha$:

$$\begin{aligned} G^\varepsilon _\alpha (t_0) < G^\varepsilon _0(t_0), \quad \text {for every}\; \alpha \in \left( 0,\frac{1}{\varepsilon }\right) , \quad G^\varepsilon _\alpha (t_0) \rightarrow 0 \quad \text {for}\; \alpha \rightarrow 1^{-}, \end{aligned}$$

for a.e. $$t_0 >0$$.

We require that the evolution of the debonding front $\ell ^\varepsilon$ follows Griffith’s criterion

2.11a $$\begin{aligned}&\dot{\ell }^\varepsilon (t)\ge 0,\end{aligned}$$

2.11b $$\begin{aligned}&G^\varepsilon _{\dot{\ell }^\varepsilon (t)}(t)\le \kappa (\ell ^\varepsilon (t)),\end{aligned}$$

2.11c $$\begin{aligned}&\dot{\ell }^\varepsilon (t)\left[ G^\varepsilon _{\dot{\ell }^\varepsilon (t)}(t)-\kappa (\ell ^\varepsilon (t))\right] =0, \end{aligned}$$

where the local toughness is assumed to be a piecewise Lipschitz and upper semicontinuous function with a finite number of discontinuities

2.12 $$\begin{aligned} \kappa :[0,+\infty )\rightarrow [c_1,c_2], \quad 0<c_1<c_2 . \end{aligned}$$

Notice that $\dot{\ell }^\varepsilon (t)$ and $G^\varepsilon _{\dot{\ell }^\varepsilon (t)}(t)$ are well defined for a.e. *t* and (2.10) gives

2.13 $$\begin{aligned} G^\varepsilon _{\dot{\ell }^\varepsilon (t)}(t) = 2\frac{1-\varepsilon \dot{\ell }^\varepsilon (t)}{1+\varepsilon \dot{\ell }^\varepsilon (t)}\dot{f}^\varepsilon (t {-}\varepsilon \ell ^\varepsilon (t))^2. \end{aligned}$$

The criterion (2.11) is derived by using the following maximum dissipation principle: for a.e. $t>0$

$$\begin{aligned} \dot{\ell }^\varepsilon (t)=\max \left\{ \alpha \in \left[ 0,\frac{1}{\varepsilon }\right) : \kappa (\ell ^\varepsilon (t))\alpha =G_\alpha ^\varepsilon (t)\alpha \right\} . \end{aligned}$$

This implies that for a.e. $t>0$, if $\dot{\ell }^\varepsilon (t)>0$, then $\kappa (\ell ^\varepsilon (t))=G^\varepsilon _{\dot{\ell }^\varepsilon (t)}(t)$, while if $\dot{\ell }^\varepsilon (t)=0$, then $\kappa (\ell ^\varepsilon (t))\ge G^\varepsilon _{\dot{\ell }^\varepsilon (t)}(t)= G^\varepsilon _0(t)$, thus (2.11) follows. Combining (2.11) with (2.13), we have an equivalent formulation of this evolution criterion. Indeed, $\ell ^\varepsilon$ satisfies Griffith’s criterion if and only if it is solution of the following Cauchy problem:

2.14 $$\begin{aligned} {\left\{ \begin{array}{ll} \displaystyle \dot{\ell }^\varepsilon (t)= \frac{1}{\varepsilon } \frac{2\dot{f}^\varepsilon (t-\varepsilon \ell ^\varepsilon (t)) ^2 - \kappa (\ell ^\varepsilon (t))}{2\dot{f}^\varepsilon (t-\varepsilon \ell ^\varepsilon (t)) ^2 + \kappa (\ell ^\varepsilon (t))}\vee 0,\\ \ell ^\varepsilon (0)=\ell _0, \end{array}\right. } \end{aligned}$$

for a.e. $$t>0$$.

The following existence and uniqueness result for the coupled problem (2.1) and (2.14) for fixed $\varepsilon >0$ was proved in Dal Maso et al. (2016, Theorem 3.5, Remark 3.6). The case of a toughness depending also on the debonding speed is addressed in the subsequent paper (Lazzaroni and Nardini 2017), where we discuss existence, uniqueness, and quasistatic limit.

### Theorem 2.3

Let $T>0$, assume (2.2), and let the local toughness $\kappa$ be as in (2.12). Then, there is a unique solution $(u^\varepsilon ,\ell ^\varepsilon ) \in C^{0,1}(\Omega ^\varepsilon _T)\times C^{0,1}([0,T])$ to the coupled problem (2.1) and (2.14). Moreover, there exists a constant $L_T^\varepsilon$ satisfying $\dot{\ell }^\varepsilon \le L_T^\varepsilon < \frac{1}{\varepsilon }$.

## A Priori Estimate and Convergence

In this section we study the limit as $\varepsilon \rightarrow 0$ of the solutions $(u^\varepsilon ,\ell ^\varepsilon )$ to the coupled problem (2.1) and (2.14). We fix $T>0$ and make the following assumptions on the data: there exists $w \in C^{0,1}([0,T])$ such that

3.1a $$\begin{aligned}&w^\varepsilon \mathop {\rightharpoonup }\limits ^{*}w \text{ weakly* } \text{ in } W^{1,\infty }(0,T),\end{aligned}$$

3.1b $$\begin{aligned}&u_0^\varepsilon \text{ is } \text{ bounded } \text{ in } W^{1,\infty }(0,\ell _0),\end{aligned}$$

3.1c $$\begin{aligned}&\varepsilon u_1^\varepsilon \text{ is } \text{ bounded } \text{ in } L^\infty (0,\ell _0). \end{aligned}$$

Notice that (3.1b) and (3.1c) imply that the initial internal energy associated with $u^\varepsilon (0,\cdot )$ is uniformly bounded with respect to $\varepsilon$.

### A Priori Bounds

We start from a uniform bound on the internal energy $\mathcal E^\varepsilon$. To this end, it is convenient to express it as in (2.9). Following Dal Maso et al. (2016, Prop. 1.14), we find the energy balance for fixed $\varepsilon >0$:

3.2 $$\begin{aligned}&\mathcal {E}^\varepsilon (t;\ell ^\varepsilon ,w^\varepsilon ) - \mathcal {E}^\varepsilon (0;\ell ^\varepsilon ,w^\varepsilon ) + \int _{\ell _0}^{\ell ^\varepsilon (t)} \kappa (x) \mathrm {d}x \nonumber \\&\quad + \int _0^t [\varepsilon \dot{w}^\varepsilon (s)-2\dot{f}^\varepsilon (s)]\dot{w}^\varepsilon (s)\mathrm {d}s =0. \end{aligned}$$

In the next proposition we derive an a priori bound for $\mathcal {E}^\varepsilon$, uniformly with respect to $\varepsilon$. First, we introduce the functions

3.3 $$\begin{aligned} \varphi ^\varepsilon (t) := t-\varepsilon \ell ^\varepsilon (t) \quad \text{ and } \quad \psi ^\varepsilon (t) := t+\varepsilon \ell ^\varepsilon (t). \end{aligned}$$

In view of Theorem 2.3, $\dot{\ell }^\varepsilon \le L_T^\varepsilon < \frac{1}{\varepsilon }$, and therefore, these functions are equi-Lipschitz. Then, we define

$$\begin{aligned} \omega ^\varepsilon (t) := \varphi ^\varepsilon ((\psi ^\varepsilon )^{-1}(t)), \end{aligned}$$

which is also equi-Lipschitz, since

$$\begin{aligned} 0<\dot{\omega }\le \frac{1-\varepsilon L_T^\varepsilon }{1+\varepsilon L_T^\varepsilon } < 1 \end{aligned}$$

for a.e. $$0\le t \le T$$.

#### Proposition 3.1

Assume (2.2), (3.1), and let $\kappa$ be as in (2.12). Then, there exists $C>0$ such that $\mathcal {E}^\varepsilon (t) \le C$ for every $\varepsilon >0$ and for every $t \in [0,T]$. Moreover, we have

3.4 $$\begin{aligned} \Vert \dot{f}^\varepsilon \Vert _{L^\infty (-\varepsilon \ell _0,T)} \le C, \end{aligned}$$

uniformly in $$\varepsilon $$.

#### Proof

We need to estimate the last term in (3.2). To this end, we notice that it is sufficient to get a uniform bound for $\dot{f}^\varepsilon$ in $L^\infty$ as in (3.4). Then the conclusion readily follows from the bounds on the initial conditions and on the toughness.

In order to obtain (3.4), we first estimate $\dot{f}^\varepsilon$ in $[-\varepsilon \ell _0, \varepsilon \ell _0]$. By differentiating (2.6) and using the assumptions (3.1), we see that

3.5 $$\begin{aligned} \mathop {{\hbox {ess sup}}}\limits _{t \in [-\varepsilon \ell _0, \varepsilon \ell _0]} |\dot{f}^\varepsilon (t)| \le \varepsilon \Vert \dot{w}^{\varepsilon }\Vert _{L^\infty (0,T)} +\frac{1}{2} \Vert \dot{u}_0^\varepsilon \Vert _{L^\infty (0,\ell _0)} + \frac{\varepsilon }{2}\Vert u_1^\varepsilon \Vert _{L^\infty (0,\ell _0)} \le C, \end{aligned}$$

for some positive constant $C>0$.

**Fig. 2:**
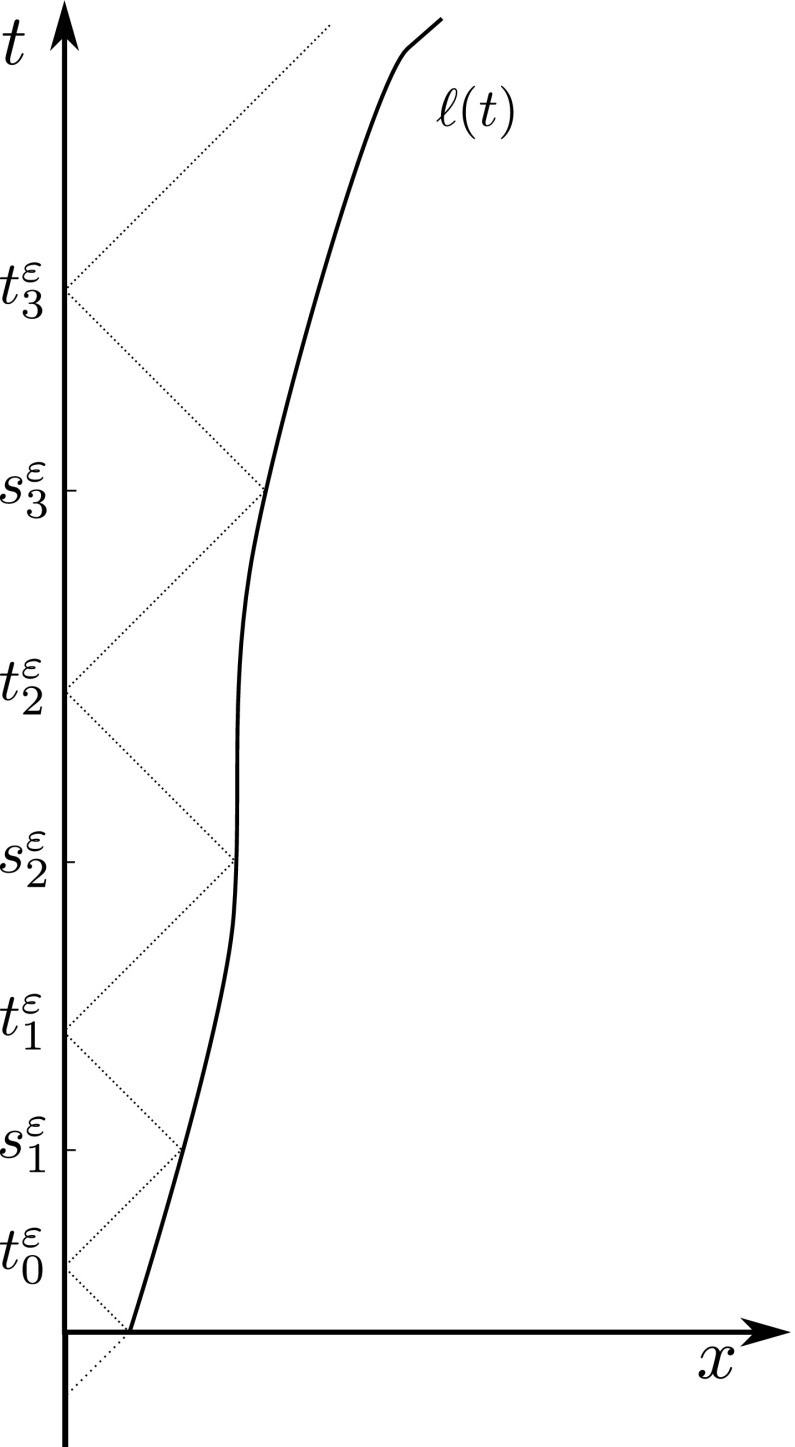
Construction of the sequences $\{s_{i}^\varepsilon \}$ and $\{t_i^\varepsilon \}$ employed in the proof of Proposition 3.1

Then, we need to extend the estimate to [0, *T*]. To this end, we mimick the construction for the existence of a solution [see Dal Maso et al. (2016, Theorem 1.8)]. More precisely, we define $t_0^\varepsilon :=\varepsilon \ell _0$ and, iteratively, $t_i^\varepsilon :=(\omega ^\varepsilon )^{-1}(t_{i-1}^\varepsilon )=\psi ^\varepsilon ((\varphi ^\varepsilon )^{-1}(t_{i-1}^\varepsilon ))$. Let also $s_{i+1}^\varepsilon :=(\varphi ^\varepsilon )^{-1}(t_i^\varepsilon )$ for $i \ge 0$. See Fig. 2.

By differentiating the “bounce formula” (2.5), we find that

3.6 $$\begin{aligned} \dot{f}^\varepsilon (t{+}\varepsilon \ell ^\varepsilon (t))= \varepsilon \dot{w}^\varepsilon (t{+}\varepsilon \ell ^\varepsilon (t)) + \frac{1-\varepsilon \dot{\ell }^\varepsilon (t)}{1+\varepsilon \dot{\ell }^\varepsilon (t)} \dot{f}^\varepsilon (t{-}\varepsilon \ell ^\varepsilon (t)). \end{aligned}$$

Then we have

$$\begin{aligned} \mathop {{\hbox {ess sup}}}\limits _{t \in \left[ t_0^\varepsilon ,t_1^\varepsilon \right] }|\dot{f}(t)| = \mathop {{\hbox {ess sup}}}\limits _{s \in \left[ 0,s_1^\varepsilon \right] } |\dot{f}^\varepsilon (s{+}\varepsilon \ell ^\varepsilon (s))| \le \varepsilon \Vert \dot{w}^\varepsilon \Vert _{L^\infty (0,T)} + \Vert \dot{f}^\varepsilon \Vert _{L^\infty (-\varepsilon \ell _0,\varepsilon \ell _0))} \le C, \end{aligned}$$

where the uniform bound follows from (3.5) up to changing the value of *C*. This implies that

$$\begin{aligned} \mathop {{\hbox {ess sup}}}\limits _{t \in \left[ -\varepsilon \ell _0,t_1^\varepsilon \right] }|\dot{f}(t)| \le C. \end{aligned}$$

We iterate this argument and use the fact that the maximum number of “bounces,” i.e. the number of times we apply formulas (2.5) and (3.6), is bounded. More precisely, there exists $N_\varepsilon$ such that $T \in (t_{N_\varepsilon }^\varepsilon ,t_{N_\varepsilon +1}^\varepsilon ]$ and, since $\ell _0>0$, we have that $N_\varepsilon \le \frac{T}{2\varepsilon \ell _0}$. Therefore,

$$\begin{aligned} \mathop {{\hbox {ess sup}}}\limits _{t\in \left[ t_{N_\varepsilon }^\varepsilon ,T\right] }|\dot{f}^\varepsilon (t)| \le&\, \mathop {{\hbox {ess sup}}}\limits _{t \in \left[ t_{N_\varepsilon }^\varepsilon ,t_{N_\varepsilon +1}^\varepsilon \right] }|\dot{f}^\varepsilon (t)| \le \varepsilon \Vert \dot{w}^\varepsilon \Vert _{L^\infty } + \mathop {{\hbox {ess sup}}}\limits _{t \in \left[ t_{N_\varepsilon -1}^\varepsilon ,t_{N_\varepsilon }^\varepsilon \right] }|\dot{f}^\varepsilon (t)| \\ \le&\, 2\varepsilon \Vert \dot{w}^\varepsilon \Vert _{L^\infty } + \mathop {{\hbox {ess sup}}}\limits _{t \in \left[ t_{N_\varepsilon -2}^\varepsilon ,t_{N_\varepsilon -1}^\varepsilon \right] }|\dot{f}^\varepsilon (t)| \le \cdots \le N_\varepsilon \varepsilon \Vert \dot{w}^\varepsilon \Vert _{L^\infty } \\&+ \,\mathop {{\hbox {ess sup}}}\limits _{t \in \left[ t_0^\varepsilon ,t_1^\varepsilon \right] }|\dot{f}^\varepsilon (t)| \\ \le&\,\frac{T}{2\ell _0} \Vert \dot{w}^\varepsilon \Vert _{L^\infty } + \mathop {{\hbox {ess sup}}}\limits _{t \in \left[ t_0^\varepsilon ,t_1^\varepsilon \right] }|\dot{f}^\varepsilon (t)| \le C. \end{aligned}$$

Then, the uniform bound on $\dot{f}^\varepsilon$ holds in $[-\varepsilon \ell _0,T]$, thus (3.4) is proved. $\square$

#### Remark 3.2

Formula (2.7a) guarantees that, for every $t\in [0,T]$, $u^\varepsilon _t(t,\cdot )$ is defined a.e. in $(0,\ell ^\varepsilon (T))$. Moreover, the uniform bound on the internal energy implies that

3.7 $$\begin{aligned} \Vert \varepsilon u^\varepsilon _t(t,\cdot )\Vert _{L^2(0,\ell ^\varepsilon (t))} \le C \quad \text{ for } \text{ every } t \in [0,T], \end{aligned}$$

where $C>0$ is independent of $\varepsilon$ and *t*.

### Convergence of the Solutions

The a priori bound on the energy allows the passage to the limit in $\ell ^\varepsilon$.

#### Proposition 3.3

Assume (2.2), (3.1), and (2.12). Let $(u^{\varepsilon },\ell ^{\varepsilon })$ be the solution to the coupled problem (2.1) and (2.14). Then, there exists $L>0$ such that $\ell ^\varepsilon (T)\le L$. Moreover, there exists a sequence $\varepsilon _k\rightarrow 0$ and an increasing function $\ell :[0,T] \rightarrow [0,L]$ such that

$$\begin{aligned} \ell ^{\varepsilon _k}(t) \rightarrow \ell (t) \end{aligned}$$

for every $$t \in [0,T]$$.

#### Proof

Since the local toughness $\kappa$ is bounded from below, a direct consequence of Proposition 3.1 is that the sequence of functions $\ell ^\varepsilon (t)$ is bounded uniformly in $\varepsilon$. Indeed, the term $-\int _0^t \dot{w}^\varepsilon (s)[\varepsilon \dot{w}(s)-2\dot{f}^\varepsilon (s)]\mathrm {d}s$ in the energy balance (3.2) is bounded, as one can see applying the Cauchy–Schwartz inequality and using (3.4). Therefore

$$\begin{aligned} \int _{\ell _0}^{\ell ^\varepsilon (t)} \kappa (x) \mathrm {d}x =- \mathcal {E}^\varepsilon (t;\ell ^\varepsilon ,w^\varepsilon ) + \mathcal {E}^\varepsilon (0;\ell ^\varepsilon ,w^\varepsilon ) - \int _0^t \dot{w}^\varepsilon (s) [\varepsilon \dot{w}^\varepsilon (s)-2\dot{f}^\varepsilon (s)] \mathrm {d}s \end{aligned}$$

is uniformly bounded. Since $\kappa \ge c_1$, it follows that there exists $C>0$ such that

3.8 $$\begin{aligned} c_1(\ell ^\varepsilon (t) - \ell _0) \le C, \end{aligned}$$

uniformly in $\varepsilon$ and for every $t \in [0,T]$. Then, using Helly’s selection principle on the sequence of uniformly bounded and increasing functions $\ell ^\varepsilon$, it is possible to extract a sequence $\ell ^{\varepsilon _k}(t)$ pointwise converging to an increasing function $\ell (t)$ for every $t \in [0,T]$. $\square$

We now prove a technical lemma stating that the graphs of $\ell ^{\varepsilon _k}$ converge to the graph of $\ell$ in the Hausdorff metric. We employ the following notation for the graph of a function:

$$\begin{aligned} \mathrm {Graph}\,\ell := \{(t,\ell (t)) :0\le t \le T\}. \end{aligned}$$

The same notation will be used for the graph of $\ell ^\varepsilon$. Since $t\mapsto \ell (t)$ may be discontinuous, we consider its extended graph

$$\begin{aligned} \mathrm {Graph}^*\ell := \{(t,x) \in [0,T]\times [0,L] :\ell (t^-) \le x \le \ell (t^+)\}, \end{aligned}$$

where $\ell (t^-)$ (resp. $\ell (t^+)$) is the left-sided (resp. right-sided) limit of $\ell$ at *t*. Given $A\subset [0,T]\times [0,L]$ and $\eta >0$ we set

$$\begin{aligned} (A)_\eta := \{(t,x) \in [0,T]\times [0,L] :d((t,x),A)< \eta \}, \end{aligned}$$

where *d* is the Euclidean distance, and we call $(A)_\eta$ the open $\eta$-neighbourhood of *A*. We also recall that, given two non-empty sets $A,B\subset [0,T]\times [0,L]$, their Hausdorff distance is defined by

$$\begin{aligned} d_{\mathcal H}(A,B) = \max \left\{ \sup _{a \in A} d(a,B), \sup _{b \in B} d(b,A)\right\} . \end{aligned}$$

Notice that

3.9 $$\begin{aligned} \text {if } d_{\mathcal H}(A,B)\le \eta , \quad \text {then } A\subset (B)_\eta \text { and } B\subset (A)_\eta . \end{aligned}$$

We say that a sequence $A_k$ converges to *A* in the sense of Hausdorff if $d_{\mathcal H}(A_k,A)\rightarrow 0$.

The Hausdorff convergence of $\mathrm {Graph}\,\ell ^{\varepsilon _k}$ to $\mathrm {Graph}^*\ell$ will be used in the proof of Theorem 3.5. To prove that $\mathrm {Graph}\,\ell ^{\varepsilon _k}$ converges to $\mathrm {Graph}^*\ell$ in the sense of Hausdorff, in the following lemma we employ the equivalent notion of Kuratowski convergence, whose definition is recalled below.

#### Lemma 3.4

The sets $\mathrm {Graph}\,\ell ^{\varepsilon _k}$ converge to $\mathrm {Graph}^*\ell$ in the sense of Hausdorff.

#### Proof

In order to prove this result we show that $\mathrm {Graph}\,\ell ^{\varepsilon _k}$ converges to $\mathrm {Graph}^*\ell$ in the sense of Kuratowski in the compact set $[0,T]\times [0,L]$. Since these sets are closed, the Kuratowski convergence implies that $\mathrm {Graph}\,\ell ^{\varepsilon _k}$ converges to $\mathrm {Graph}^*\ell$ in the sense of Hausdorff, cf. Ambrosio and Tilli (2004, Proposition 4.4.14). We recall that $\mathrm {Graph}\,\ell ^{\varepsilon _k}$ converges to $\mathrm {Graph}^*\ell$ in the sense of Kuratowski if the following two conditions are both satisfied:

(i) Let $(t,x) {\in } [0,T] \times [0,L]$ and let $(t_k,x_k) \in \mathrm {Graph}\,\ell ^{\varepsilon _k}$ be a sequence such that $(t_{k_n},x_{k_n}) \rightarrow (t,x)$ for some subsequence. Then, $(t,x) \in \mathrm {Graph}^*\ell$.
(ii) For every $(t,x) \in \mathrm {Graph}^*\ell$ there exists a whole sequence such that $(t_k,x_k) \in \mathrm {Graph}\,\ell ^{\varepsilon _k}$ and $(t_k,x_k) \rightarrow (t,x)$.

We prove condition (i) arguing by contradiction. Let thus $(t,x) \in [0,T]\times [0,L]$ and $(t_k,x_k) \in \mathrm {Graph}\,\ell ^{\varepsilon _k}$ be such that $(t_k,x_k) \rightarrow (t,x)$ up to a subsequence (not relabelled) and assume that $(t,x) \notin \mathrm {Graph}^*\ell$, i.e. $x \notin [\ell (t^-),\ell (t^+)]$. We consider the case where $x < \ell (t^-)$, the case $x > \ell (t^+)$ being analogous. By assumption, there exists $k_0 \in \mathbb {N}$ such that for every $k \ge k_0$ we have $\ell ^{\varepsilon _k}(t_k) < \ell (t^-)$. By the definition of $\ell (t^-)$ and the monotonicity of $\ell$, there exists $\eta > 0$ such that $\ell ^{\varepsilon _k}(t_k) < \ell (t{-}\eta )$ for every $k \ge k_0$. For *k* large, we have $t_k > t-\eta$. Therefore, by the monotonicity of $\ell ^{\varepsilon _k}$, we get

$$\begin{aligned} \ell ^{\varepsilon _k}(t-\eta ) \le \ell ^{\varepsilon _k}(t_k) < \ell (t-\eta ), \end{aligned}$$

which leads to contradiction, by the pointwise convergence of $\ell ^{\varepsilon _k}(t-\eta )$.

We now prove condition (ii). Let $(t,x) \in \mathrm {Graph}^*\ell$. Then, for every $\eta >0$ we have $\ell (t-\eta ) \le x \le \ell (t+\eta )$. We claim that there is a sequence $x_k \rightarrow x$ such that $x_k \in [\ell ^{\varepsilon _k}(t{-}\eta ),\ell ^{\varepsilon _k}(t{+}\eta )]$. Specifically, if $\ell (t{-}\eta )< x < \ell (t{+}\eta )$ we take $x_k:=x$; if $x= \ell (t{-}\eta )$ we take $x_k:= \ell ^{\varepsilon _k}(t{-}\eta )$; if $x = \ell (t{+}\eta )$ we take $x_k := \ell ^{\varepsilon _k}(t{+}\eta )$; in each case by pointwise convergence we conclude that $x_k\rightarrow x$. Then, by continuity and monotonicity of $\ell ^{\varepsilon _k}$, there exists $t_k \in [t{-}\eta ,t{+}\eta ]$ such that $\ell ^{\varepsilon _k}(t_k)=x_k$. We conclude by the arbitrariness of $\eta$. $\square$

We now investigate on the limit behaviour of $u^\varepsilon$. The next theorem shows that the limit displacement solves problem (1.5).

#### Theorem 3.5

Assume (2.2), (3.1), and (2.12). Let $(u^{\varepsilon },\ell ^{\varepsilon })$ be the solution to the coupled problem (2.1) and (2.14). Let *L* and $\varepsilon _k$ be as in Proposition 3.3. Then,

3.10 $$\begin{aligned} u^{\varepsilon _k} \rightharpoonup u \text{ weakly } \text{ in } L^2(0,T;H^1(0,L)), \end{aligned}$$

where

3.11 $$\begin{aligned} u(t,x) = {\left\{ \begin{array}{ll} -\frac{w(t)}{\ell (t)}x + w(t) &{}\; \text {for a.e. } (t,x):x<\ell (t), \\ 0 &{} \;\text {for a.e. } (t,x):x\ge \ell (t) . \end{array}\right. } \end{aligned}$$

#### Proof

We recall that $u^{\varepsilon _k}(t,x)=0$ whenever $x>\ell ^{\varepsilon _k}(T)$. By Proposition 3.1 and by (2.8), $u_x^{\varepsilon _k}$ is bounded in $L^\infty (0,T;L^2(0,L))$ and therefore in $L^2(0,T;L^2(0,L))$ as well. We can thus extract a subsequence (not relabelled) and find a function $q \in L^2(0,T;L^2(0,L))$ such that

3.12 $$\begin{aligned} u_x^{\varepsilon _k} \rightharpoonup q \text{ in } L^2(0,T;L^2(0,L)). \end{aligned}$$

We have

3.13 $$\begin{aligned} u^{\varepsilon _k} (t,x) = w^\varepsilon (t) + \int _0^x u_x^{\varepsilon _k}(t,\xi )\mathrm {d}\xi , \end{aligned}$$

for every $(t,x) \in \Omega _T^{\varepsilon _k}$. In particular, $u^{\varepsilon _k}$ is bounded in $L^2(0,T;L^2(0,L))$ and (up to extracting a further subsequence, not relabelled) there exists $u\in L^2(0,T;L^2(0,L))$ such that

3.14 $$\begin{aligned} u^{\varepsilon _k} \rightharpoonup u \text{ in } L^2(0,T;L^2(0,L)). \end{aligned}$$

We remark that at this stage of the proof the limit displacement *u* may depend on the subsequence extracted in (3.14); however, at the end of the proof we shall show the explicit formula (3.11), which implies that the limit is the same on the whole sequence $\varepsilon _k$ extracted in Proposition 3.3.

We now pick a function $p(t,x) \in L^2(0,T;L^2(0,L))$ and integrate (3.13) over $(0,T)\times (0,L)$. By the Fubini Theorem we obtain

$$\begin{aligned}&\int _0^T\!\!\!\!\int _0^L u^{\varepsilon _k}(t,x) p(t,x) \mathrm {d}x \mathrm {d}t\\&\quad = \int _0^T\!\!\!\!\int _0^L w^\varepsilon (t) p(t,x)\mathrm {d}x \mathrm {d}t + \int _0^T \!\!\!\!\int _0^L p(t,x)\left( \int _0^x u^{\varepsilon _k}_x(t,\xi )\mathrm {d}\xi \right) \mathrm {d}x \mathrm {d}t \\&\quad = \int _0^T\!\!\!\!\int _0^L w^\varepsilon (t) p(t,x)\mathrm {d}x \mathrm {d}t + \int _0^T \!\!\!\!\int _0^L\!\!\left( \int _\xi ^L p(t,x) \mathrm {d}x\right) u^{\varepsilon _k}_x(t,\xi )\mathrm {d}\xi \mathrm {d}t \\&\quad = \int _0^T\!\!\!\!\int _0^L w^\varepsilon (t) p(t,x)\mathrm {d}x \mathrm {d}t + \int _0^T \!\!\!\!\int _0^L u^{\varepsilon _k}_x(t,\xi ) P(t,\xi )\mathrm {d}\xi \mathrm {d}t, \end{aligned}$$

where $P(t,\xi ) = \int _\xi ^L p(t,x) \mathrm {d}x$ is still in $L^2(0,T;L^2(0,L))$ by the Jensen inequality. Using (3.1a), (3.12), and (3.14), we find

$$\begin{aligned} u(t,x) = w(t) + \int _0^x q(t,\xi ) \mathrm {d}\xi , \end{aligned}$$

for a.e. $(t,x)\in (0,T)\times (0,L)$. This shows that $q=u_x$. We thus have proved that $u^{\varepsilon _k} \rightharpoonup u$ in $L^2(0,T;H^1(0,L))$.

We now prove (3.11). We employ Lemma 3.4, which can be rephrased as follows by using the open $\eta$-neighbourhood of $\mathrm {Graph}^*\ell$ and (3.9): for every $\eta \in (0, \ell _0)$ and for *k* sufficiently large we have

**Fig. 3:**
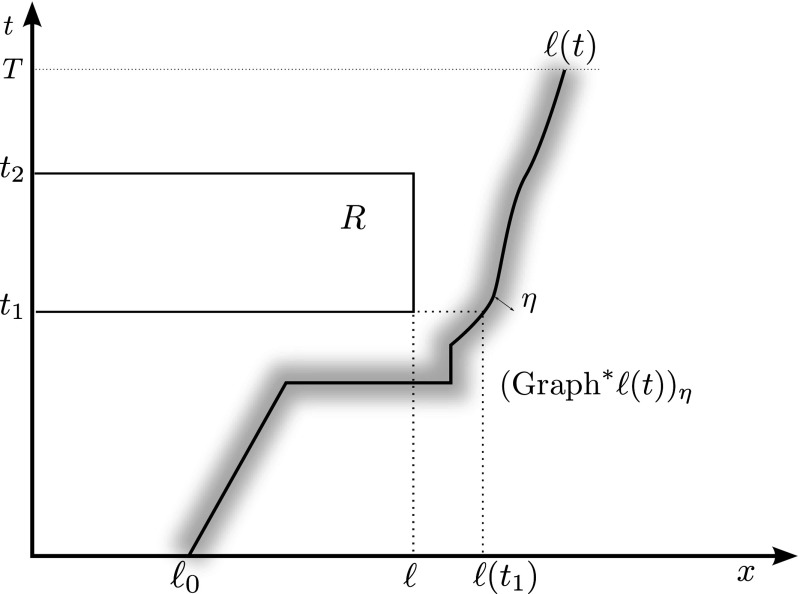
The set $(\mathrm {Graph}^*\ell )_\eta$ and the rectangle *R* employed in the proof of Theorem 3.5

3.15 $$\begin{aligned} \mathrm {Graph}\,\ell ^{\varepsilon _k} \subset (\mathrm {Graph}^*\ell )_\eta , \end{aligned}$$

see Fig. 3. Hence, we pick a test function $v \in H^1((0,T)\times (0,L))$ such that $v(t,0)=0$ and $v(t,x)=0$ whenever $(t,x) \in (\mathrm {Graph}^*\ell )_\eta$. By integration by parts in time and space, the equation solved by $u^{\varepsilon _k}$ gives

3.16 $$\begin{aligned} 0=&\int _0^T \int _0^{L} \left( \varepsilon _k^2 u^{\varepsilon _k}_{tt} - u^{\varepsilon _k}_{xx}\right) v \mathrm {d}x \mathrm {d}t \nonumber \\ =&-\int _0^T \int _0^{L} \left( \varepsilon _k^2 u^{\varepsilon _k}_t v_t - u^{\varepsilon _k}_x v_x\right) \mathrm {d}x \mathrm {d}t +\varepsilon _k^2 \int _0^{L} u^{\varepsilon _k}_t(T,x)v(T,x) \mathrm {d}x \nonumber \\&-\varepsilon _k^2\int _0^{\ell _0} u_1(x) v(0,x) \mathrm {d}x -\varepsilon _k^2 \int _{\ell _0}^{L}u^{\varepsilon _k}_t((\ell ^{\varepsilon _k})^{-1}(x), x) v((\ell ^{\varepsilon _k})^{-1}(x), x) \mathrm {d}x \nonumber \\&- \int _0^T u^{\varepsilon _k}_x(t,\ell ^{\varepsilon _k}(t))v(t,\ell ^{\varepsilon _k}(t)) \mathrm {d}t + \int _0^T u^{\varepsilon _k}_x(t,0)v(t,0)\mathrm {d}t. \end{aligned}$$

Notice that the boundary term in the last expression makes sense since $(\ell ^{\varepsilon _k})^{-1}(x)$ is defined for a.e. $x\in [0,L]$.

We now show that each summand in (3.16) converges to zero as $k\rightarrow \infty$. Using (3.7) we obtain

$$\begin{aligned} \varepsilon _k^2 \int _0^{L} u^{\varepsilon _k}_t(T,x)v(T,x)\mathrm {d}x \le \varepsilon _k \Vert \varepsilon _k u^{\varepsilon _k}_t (T,\cdot )\Vert _{L^2(0,L)}\Vert v(T,\cdot )\Vert _{L^2(0,L)} \rightarrow 0 . \end{aligned}$$

Integrating (3.7) in time we find that $-\int _0^T \int _0^{L} \varepsilon _k^2 u^{\varepsilon _k}_t v_t \mathrm {d}x \mathrm {d}t\rightarrow 0$. Moreover,

$$\begin{aligned} -\varepsilon _k^2\int _0^{\ell _0} u_1(x) v(0,x) \mathrm {d}x \rightarrow 0, \end{aligned}$$

since $\varepsilon _k u_1$ is bounded by (3.1c). We also notice that

$$\begin{aligned} \varepsilon _k^2&\int _{\ell _0}^{\ell ^{\varepsilon _k}(T)}\!\!\!u^{\varepsilon _k}_t((\ell ^{\varepsilon _k})^{-1}(x), x) v((\ell ^{\varepsilon _k})^{-1}(x), x) \mathrm {d}x \\&\quad = -\varepsilon _k^2 \int _0^T\!\!\! u^{\varepsilon _k}_t(t,\ell ^{\varepsilon _k}(t))v(t,\ell ^{\varepsilon _k}(t)) \dot{\ell }^{\varepsilon ^\varepsilon _k}(t) \mathrm {d}t = 0, \end{aligned}$$

since $v(t,x)=0$ in $(\mathrm {Graph}^*\ell )_\eta$ and (3.15). Finally,

$$\begin{aligned} \int _0^T u^{\varepsilon _k}_x(t,0)v(t,0)\mathrm {d}t = 0 \end{aligned}$$

by assumption on *v*. This implies that in the limit we find

3.17 $$\begin{aligned} \int _0^T \int _0^{\ell (t)} u_x v_x=0, \end{aligned}$$

for every test function *v* in $H^1((0,T)\times (0,L))$ such that $v(t,0)=0$ and $v(t,x)=0$ whenever $(t,x) \in (\mathrm {Graph}^*\ell )_\eta$.

Finally, we prove that the limit function $u(t,\cdot )$ is affine in $[0,\ell (t)]$ for every *t*. We fix a rectangle $R:=(t_1,t_2)\times (0,\ell )$, with $t_1,t_2 \in [0,T]$ and $0<\ell <\ell (t_1)$, see Fig. 3. Let *v* be of the form $v(t,x) = \alpha (t) \beta (x)$, with $\alpha \in H^1_0(t_1,t_2)$ and $\beta \in H^1_0(0,\ell )$. Then, by (3.17) we know that

$$\begin{aligned} \int _{t_1}^{t_2} \alpha (t) \left( \int _0^\ell u_x(t,x) \dot{\beta }(x) \mathrm {d}x\right) \mathrm {d}t = 0. \end{aligned}$$

Applying twice the Fundamental Lemma of Calculus of Variations, we find *a*(*t*) and *b*(*t*) such that

3.18 $$\begin{aligned} u(t,x) = a(t)x+b(t), \end{aligned}$$

for a.e. $(t,x) \in R$. Then, by the arbitrariness of *R*, Eq. (3.18) is satisfied almost everywhere in $\{(t,x):x<\ell (t)\}$.

On the other hand, in the region $\{(t,x):x\ge \ell (t)\}$ we have $u(t,x)=u^{\varepsilon _k}(t,x)=0$. Then we obtain the boundary condition $u(t,\ell (t))=0$ for a.e. *t*. By the weak convergence of $u^{\varepsilon _k}$ to *u* and by (3.1a) we also recover the boundary condition $u(t,0)=w(t)$ for every *t*. This implies, together with (3.18), that

$$\begin{aligned} u(t,x) = -\frac{w(t)}{\ell (t)}x + w(t), \end{aligned}$$

for a.e. $t \in [0,T]$ and a.e. $x \in [0,\ell (t)]$, while $u=0$ for $x > \ell (t)$. $\square$

### Convergence of the Stability Condition

At this stage of the asymptotic analysis we have found a limit pair $(u,\ell )$ that describes the evolution of the debonding when the speed of the external loading tends to zero. We now investigate on the limit of Griffith’s criterion (2.11) and we question whether the limit pair $(u,\ell )$ satisfies the quasistatic version of this criterion, i.e. whether $(u,\ell )$ is a rate-independent evolution according to the definition below.

Given a non-decreasing function $\lambda :[0,T]\rightarrow [0,L]$ and an external loading $w\in {C}^{0,1}([0,T])$ as above, for every $t\in [0,T]$ the internal quasistatic (potential) energy governing the process is

$$\begin{aligned} \mathcal {E}_\mathrm{qs}(t;\lambda ,w):= \min \left\{ \frac{1}{2} \int _0^{\lambda (t)} \dot{v}(x)^2\mathrm {d}x : v\in H^1(0,L), v(0)=w(t), v(\lambda (t))=0 \right\} , \end{aligned}$$

where $\dot{v}$ denotes the derivative of *v* with respect to *x*, as always in this paper for functions of only one variable. As in Sect. 2, we define the quasistatic energy release rate $G_\mathrm{qs}$ as the opposite of the derivative of $\mathcal {E}_\mathrm{qs}(t;\lambda ,w)$ with respect to $\lambda$, i.e.

$$\begin{aligned} G_\mathrm{qs}(t):=- \partial _\lambda \mathcal {E}_\mathrm{qs}(t;\lambda ,w) . \end{aligned}$$

Notice that $\partial _\lambda$ has to be interpreted as a Gâ teaux differential with respect to the function $\lambda$, indeed the displacement *u* depends on $\lambda$. The expression of $G_\mathrm{qs}(t)$ is simplified by taking into account that an equilibrium displacement is affine in $(0,\lambda (t))$, see Remark 3.7.

#### Definition 3.6

(*Rate-independent evolution*) Let $\lambda :[0,T]\rightarrow [0,L]$ be a non-decreasing function and $v\in L^2(0,T;H^1(0,L))$. We say that $(v,\lambda )$ is a rate-independent evolution if it satisfies the equilibrium equation for a.e. $t\in [0,T]$,

3.19a $$\begin{aligned}&v_{xx}(t,x)=0,&\text { for } 0<x<\lambda (t), \end{aligned}$$

3.19b $$\begin{aligned}&v(t,0)=w(t), \end{aligned}$$

3.19c $$\begin{aligned}&v(t,x)=0,&\text { for } x\ge \lambda (t), \end{aligned}$$

and the quasistatic formulation of Griffith’s criterion for a.e. $t>0$,

3.20a $$\begin{aligned}&\dot{\lambda }(t)\ge 0,\end{aligned}$$

3.20b $$\begin{aligned}&G_\mathrm{qs}(t)\le \kappa (\lambda (t)),\end{aligned}$$

3.20c $$\begin{aligned}&\left[ G_\mathrm{qs}(t)-\kappa (\lambda (t))\right] \dot{\lambda }(t)=0. \end{aligned}$$

#### Remark 3.7

By (3.19), we know that $v(t,x)=\big [-\frac{w(t)}{\lambda (t)}x+w(t)\big ] \vee 0$ for a.e. $t \in [0,T]$. Then, the quasistatic energy release rate can be explicitly computed and is given by

$$\begin{aligned} G_\mathrm{qs}(t)= \frac{w(t)^2}{2\lambda (t)^2}=\frac{1}{2}v_x(t,\lambda (t))^2. \end{aligned}$$

Moreover, under the additional assumption that $\lambda \in AC([0,T])$, (3.20c) is equivalent to the energy-dissipation balance that reads as follows:

3.21 $$\begin{aligned} \mathcal {E}_\mathrm{qs}(t;\lambda ,w)-\mathcal {E}_\mathrm{qs}(0;\lambda ,w) + \int _{\lambda _0}^{\lambda (t)} \kappa (x) \mathrm {d}x + \int _0^t v_x(s,0)\dot{w}(s) \mathrm {d}s = 0, \end{aligned}$$

for every $t \in [0,T]$. Indeed, we use again the formula for *v* and differentiate (3.21) with respect to *t*, obtaining for a.e. $t\in [0,T]$

$$\begin{aligned} \frac{w(t)\dot{w}(t)}{\lambda (t)} + \dot{\lambda }(t) \left[ -\frac{w(t)^2}{2\lambda (t)^2}+\kappa (\lambda (t))\right] -\frac{w(t)\dot{w}(t)}{\lambda (t)}=0, \end{aligned}$$

which is (3.20c). Therefore, Definition 3.6 complies with the usual notion of rate-independent evolution satisfying a first-order stability and an energy-dissipation balance, see Mielke and Roubíček (2015).

Notice that (3.19) and (3.20) do not prescribe the behaviour of the system at time discontinuities. In order to determine suitable solutions, additional requirements can be imposed, e.g. requiring that the total energy is conserved also after jumps in time.

#### Remark 3.8

By (3.20c), we deduce that three different regimes for the evolution of a rate-independent debonding front $\lambda$ are possible: $\lambda$ is constant in a time subinterval, or it has a jump, or it is of the form

$$\begin{aligned} \lambda (t) = \frac{w(t)}{\sqrt{2\kappa (\lambda (t))}}. \end{aligned}$$

Notice that, in the case of a non-decreasing local toughness $\kappa$, the quasistatic energy functional

$$\begin{aligned} \mathcal {E}_\mathrm{qs}(\lambda ) = \frac{w}{2\lambda } + \int _0^\lambda \kappa (x) \mathrm {d}x \end{aligned}$$

is convex. This implies that a rate-independent evolution is a global minimizer for the total quasistatic energy.

We now consider the pair $(u,\ell )$ obtained in Proposition 3.3 and Theorem 3.5. We want to verify if $(u,\ell )$ satisfies Definition 3.6. First, we observe that by construction (cf. the application of Helly’s theorem in Proposition 3.3) $t \mapsto \ell (t)$ is non-decreasing, thus (3.20a) automatically holds for a.e. *t*.

Next, we show that (3.20b) is satisfied. We first prove a few technical results.

#### Lemma 3.9

Let $\Omega$ be a bounded domain in $\mathbb {R}^N$ and $g_n \rightarrow 1$ in measure, with $g_n:\Omega \rightarrow \mathbb {R}$ equibounded. Then, $g_n \rightarrow 1$ strongly in $L^2(\Omega )$.

#### Proof

Fix $\eta , \delta >0$. By the convergence in measure of the sequence $g_n$, there exists $n_0 \in \mathbb {N}$ and a set

$$\begin{aligned} A_\delta := \{x :|g_n - 1| >\delta \} \end{aligned}$$

such that $|A_\delta |<\eta$ for every $n>n_0$. Therefore,

$$\begin{aligned} \int _\Omega |g_n - 1|^2 \mathrm {d}x&= \int _{A_\delta } |g_n-1|^2\mathrm {d}x + \int _{\Omega {\setminus }A_\delta } |g_n-1|^2\mathrm {d}x \\&\le C\int _{A_\delta } \mathrm {d}x + \int _\Omega \delta ^2 \mathrm {d}x, \end{aligned}$$

where $C>0$. In the last passage we have used the equiboundedness of $g_n$. The arbitrariness of $\eta$ and $\delta$ leads to the conclusion of the proof. $\square$

#### Lemma 3.10

Let $\Omega$ be a bounded open interval, $g_n :\Omega \rightarrow \mathbb {R}$ a sequence of functions such that $g_n \rightarrow 1$ in measure and let $\rho _n :\Omega \rightarrow \Omega$ such that $\rho _n^{-1}$ are equi-Lipschitz and $\rho _n \rightarrow 1$ uniformly in $\overline{\Omega }$. Then, $g_n \circ \rho _n \rightarrow 1$ in measure.

#### Proof

For every $\delta > 0$ we have

$$\begin{aligned} \{x :|g_n \circ \rho _n -1 |>\delta \} = \rho _n^{-1} \left( \{y :|g_n(y) -1|>\delta \} \right) . \end{aligned}$$

Since $\rho _n^{-1}$ is equi-Lipschitz,

$$\begin{aligned} |\rho _n^{-1} \{y :|g_n(y) -1|>\delta \}| \le C |\{y :|g_n(y) -1|>\delta \}|, \end{aligned}$$

where *C* is a positive constant. We conclude using the convergence in measure of $g_n$ to 1. $\square$

#### Theorem 3.11

Assume (2.2), (2.12), and (3.1) and let $(u^{\varepsilon },\ell ^{\varepsilon })$ be the solution to the coupled problem (2.1) and (2.14). Let *L* and $\varepsilon _k$ be as in Proposition 3.3. Then, for a.e. $t \in [0,T]$ conditions (3.20a) and (3.20b) are satisfied.

#### Proof

By (3.4) $\dot{f}^{\varepsilon _k}$ is bounded in $L^\infty (-\varepsilon _k\ell _0,T)$ uniformly with respect to $\varepsilon _k$. Therefore, $\dot{f}^{\varepsilon _k}$ is bounded in $L^2(-\varepsilon _k\ell _0,T)$ as well. Since $f^{\varepsilon _k}(0)=0$, we have that $f^{\varepsilon _k}$ is bounded in $H^1(-\varepsilon _k\ell _0,T)$ and thus, up to a subsequence (not relabelled), $f^{\varepsilon _k}$ weakly converges to a function *f* in $H^1(0,T)$. Moreover, it is possible to characterise the limit function *f* in terms of *w* and $\ell$. If we differentiate (2.4) with respect to *x* we find

$$\begin{aligned} u^\varepsilon _x(t,x) =-\dot{f}^{\varepsilon _k}(t{-}\varepsilon _k x) + \varepsilon _k \dot{w}^\varepsilon _k (t{+}\varepsilon _k x) - \dot{f}^{\varepsilon _k} (t{+}\varepsilon _k x). \end{aligned}$$

By (3.10) and (3.11), we know that, up to a subsequence,

$$\begin{aligned} u^{\varepsilon _k}_x \rightharpoonup -\frac{w}{\ell }\quad \text{ in } L^2(0,T;L^2(0,L)). \end{aligned}$$

For every $p \in L^2(0,T)$ we have

$$\begin{aligned}&\lim _{k \rightarrow \infty } \int _0^L \int _0^T u^{\varepsilon _k}_x(t,x) p(t) \mathrm {d}t \mathrm {d}x \\&\quad = -\lim _{k \rightarrow \infty } \int _0^L \int _0^T \left[ \dot{f}^{\varepsilon _k}(t{-}\varepsilon _k x) + \dot{f}^{\varepsilon _k}(t{+}\varepsilon _k x)\right] p(t)\mathrm {d}t \mathrm {d}x \\&\quad = -\lim _{k \rightarrow \infty }\int _0^L \int _{-\varepsilon _kx}^{T-\varepsilon _k x} \dot{f}^{\varepsilon _k}(s)p(s+\varepsilon _kx)\mathrm {d}s \mathrm {d}x \\&\qquad -\lim _{k \rightarrow \infty }\int _0^L \int _{\varepsilon _kx}^{T+\varepsilon _k x} \dot{f}^{\varepsilon _k}(s)p(s-\varepsilon _kx)\mathrm {d}s \mathrm {d}x \\&\quad =-\int _0^L \int _0^T 2\dot{f}(t)p(t) \mathrm {d}t\mathrm {d}x, \end{aligned}$$

by the continuity in $L^2$ with respect to translations and the weak convergence of $\dot{f}^{\varepsilon _k}$. Therefore,

3.22 $$\begin{aligned} \dot{f}(t) = \frac{w(t)}{2\ell (t)} \quad \text {for a.e. } t \in [0,T]. \end{aligned}$$

Since $f^{\varepsilon _k}(0)=0$, we have $f(0)=0$. Therefore,

$$\begin{aligned} f(t) = \int _0^t \frac{w(s)}{2\ell (s)} \mathrm {d}s. \end{aligned}$$

We now use Griffith’s condition (2.11b) and (2.13) in order to find that, for every subinterval $(a,b) \subset (0,T)$,

3.23 $$\begin{aligned} \int _a^b \sqrt{\kappa (\ell ^{\varepsilon _k}(t))} \mathrm {d}t \ge \int _a^b \sqrt{G^{\varepsilon _k}(t)} \mathrm {d}t = \int _a^b \sqrt{2g_{\varepsilon _k}(t)} \dot{f}^{\varepsilon _k}(\varphi ^\varepsilon _k(t)) \mathrm {d}t, \end{aligned}$$

where $g_{\varepsilon _k}(t) := \frac{1-\varepsilon _k \dot{\ell }^{\varepsilon _k}(t)}{1+\varepsilon _k \dot{\ell }^{\varepsilon _k}(t)}$ and $\varphi ^\varepsilon _k(t)$ is as in (3.3). Since $\dot{\varphi }^{\varepsilon _k}(t)\le 1$, we can continue (3.23) and find that

3.24 $$\begin{aligned} \int _a^b \sqrt{G^{\varepsilon _k}(t)} \mathrm {d}t&\ge \int _a^b \sqrt{2g_{\varepsilon _k}(t)} \dot{f}^{\varepsilon _k}(\varphi ^{\varepsilon _k}(t)) \dot{\varphi }^{\varepsilon _k}(t) \mathrm {d}t \nonumber \\&= \int _{-\varepsilon _k\ell _0}^T \mathbbm {1}_{(\varphi ^{\varepsilon _k}(a),\varphi ^{\varepsilon _k}(b))}(s) \sqrt{2g_{\varepsilon _k}((\varphi ^{\varepsilon _k})^{-1}(s))}\dot{f}^{\varepsilon _k}(s)\mathrm {d}s. \end{aligned}$$

By Čebyšëv’s inequality and by the fact that, by (3.8), $\ell ^{\varepsilon _k}$ is uniformly bounded, we now show that $\varepsilon \dot{\ell }^{\varepsilon _k} \rightarrow 0$ in measure. Indeed, for every $\eta >0$ there exists a constant $C=C(\eta )>0$ such that

$$\begin{aligned} \left| \{t\in [0,T] :\varepsilon _k \dot{\ell }^{\varepsilon _k}(t) >\eta \}\right| \le \frac{1}{\eta } \varepsilon _k\int _0^T\dot{\ell }^{\varepsilon _k}(t)\mathrm {d}t \le \varepsilon _k C. \end{aligned}$$

This implies that $g_{\varepsilon _k}$ converges in measure to one. Since $\varphi ^{\varepsilon _k}$ is equi-Lipschitz, then Lemma 3.10 ensures that $g_{\varepsilon _k} \circ (\varphi ^{\varepsilon _k})^{-1} \rightarrow 1$ in measure. By Lemma 3.9, $g_{\varepsilon _k} ((\varphi ^{\varepsilon _k})^{-1})\rightarrow 1$ strongly in $L^2(0,T)$. Finally, since $\mathbbm {1}_{(\varphi ^{\varepsilon _k}(a),\varphi ^{\varepsilon _k}(b))}$ strongly converges to $\mathbbm {1}_{(a,b)}$ in $L^2(0,T)$ (because $\varphi ^{\varepsilon _k}(t) \rightarrow t$ uniformly) and since $\dot{f}^{\varepsilon _k} \rightharpoonup \dot{f}$ in $L^2(0,T)$, then the right-hand side of (3.24) tends to

$$\begin{aligned} \int _a^b \sqrt{2} \dot{f}(s) \mathrm {d}s = \int _a^b \sqrt{G_\mathrm{qs}(t)} \mathrm {d}s \end{aligned}$$

as $k \rightarrow \infty$, where the equality follows by (3.22). Therefore,

$$\begin{aligned} \int _a^b \sqrt{G_\mathrm{qs}(t)}\mathrm {d}t \le \limsup _k \int _a^b \sqrt{\kappa (\ell ^{\varepsilon _k}(t)} \mathrm {d}t. \end{aligned}$$

By the Fatou lemma and by upper semicontinuity of $\kappa$, we find that

$$\begin{aligned} \limsup _k \int _a^b \sqrt{\kappa (\ell ^{\varepsilon _k}(t))} \mathrm {d}t \le \int _a^b \limsup _k \sqrt{\kappa (\ell ^{\varepsilon _k}(t))} \mathrm {d}t \le \int _a^b \sqrt{\kappa (\ell (t))}\mathrm {d}t. \end{aligned}$$

Using the arbitrariness of (*a*, *b*), we obtain

$$\begin{aligned} G_\mathrm{qs}(t) = \frac{w(t)^2}{2\ell (t)^2}=2\dot{f}(t)^2 \le \kappa (\ell (t)) \end{aligned}$$

for a.e. $t \in [0,T]$, thus (3.20b) is proved. $\square$

#### Remark 3.12

We recall that Theorem 3.5 guarantees only that $u^{\varepsilon _k}$ converges to *u* weakly in $L^2(0,T;H^1(0,L))$. If in addition we knew that

3.25 $$\begin{aligned} u^{\varepsilon _k}(t,\cdot ) \rightharpoonup u(t,\cdot ) \text { weakly in } H^1(0,L) \text { for every } t\in [0,T], \end{aligned}$$

then it would be possible also to pass to the limit in the activation condition (2.11c) obtaining (3.20c).

To this end, besides (3.1) we assume that $w^{\varepsilon _k}$ converges to *w* strongly in $H^1(0,T)$, that $u_0^{\varepsilon _k}$ converges to $u_0$ strongly in $H^1(0,\ell _0)$, and that $\varepsilon u_1$ converges to 0 strongly in $L^2(0,\ell _0)$, i.e. the initial kinetic energy tends to zero. Then, by (3.25) the lower semicontinuity of the potential energy ensures that $\mathcal E_\mathrm{qs}(t;\ell ,w)\le \liminf _{k\rightarrow \infty }\mathcal E^{\varepsilon _k}(t;\ell ^{\varepsilon _k},w^{\varepsilon _k})$. Passing to the limit in (3.2) and using (3.22), we obtain an energy inequality; the opposite inequality derives from (3.20b) with arguments similar to Remark 3.7. We thus obtain (3.21) which is equivalent to the activation condition (at least in time intervals with no jumps).

However, conditions (3.25) and (3.21) may not hold in general, as shown in the example of the following section. The example shows that in general (2.11c) does not pass to the limit and (3.20c) is not satisfied, even in the case of a constant toughness.

## Counterexample to the Convergence of the Activation Condition

We now show an explicit case where the convergence of (2.11c)–(3.20c) fails.

A first counterexample to the convergence of the activation condition was presented in Lazzaroni et al. (2012). In this case, the singular behaviour is due to the choice of a toughness with discontinuities. More precisely, in Lazzaroni et al. (2012) it is assumed that $\kappa (x)=\kappa _1$ in $(\ell _1,\ell _1+\delta )$ and $\kappa (x)=\kappa _2$ for $x\notin (\ell _1,\ell _1+\delta )$, where $\kappa _1<\kappa _2$, $\ell _1>\ell _0$, and $\delta$ is sufficiently small; this models a short defect of the glue between the film and the substrate.

In this section we show an example of singular behaviour arising even if the local toughness is constant. For simplicity we set $\kappa :=\frac{1}{2}$. Moreover, we fix the external loading

$$\begin{aligned} w^\varepsilon (s) := s +2\left( \sqrt{1+\varepsilon ^2}-\varepsilon \left\lfloor \frac{1}{\varepsilon }\right\rfloor \right) \end{aligned}$$

and the initial conditions

4.1a $$\begin{aligned}&\ell _0:=2, \quad u_1^\varepsilon (x):=1,\nonumber \\&u_0^\varepsilon (x):={\left\{ \begin{array}{ll} \left( 2\varepsilon \left\lfloor \frac{1}{\varepsilon }\right\rfloor -\sqrt{1+\varepsilon ^2}\right) x+2\left( \sqrt{1+\varepsilon ^2}-\varepsilon \left\lfloor \frac{1}{\varepsilon }\right\rfloor \right) , &{} \;0\le x\le 1,\\ -\sqrt{1+\varepsilon ^2}x+2\sqrt{1+\varepsilon ^2}, &{} \;1 \le x \le 2. \end{array}\right. }\nonumber \\ \end{aligned}$$

**Fig. 4:**
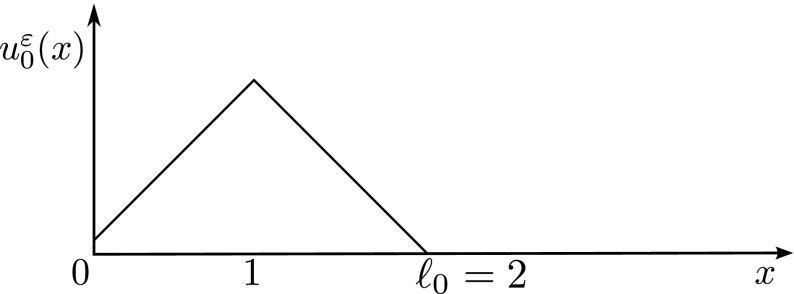
The initial datum $u_0^\varepsilon$ in the example of Sect. 4. We have $u_0^\varepsilon (0)=2(\sqrt{1+\varepsilon ^2}-\varepsilon \lfloor \frac{1}{\varepsilon }\rfloor )$, $u_0^\varepsilon (1)=\sqrt{1+\varepsilon ^2}$, and $u_0^\varepsilon (2)=0$

Here $\lfloor \cdot \rfloor$ denotes the integer part. Notice that $w^\varepsilon$ is a perturbation of $w(s):=s$, $u_0^\varepsilon$ is a perturbation of a “hat function” $u_0(x):=x \wedge (2-x)$, and (2.2b) is satisfied; see Fig. 4. Moreover, the initial kinetic energy $\frac{1}{2}\Vert \varepsilon u^\varepsilon _1\Vert ^2_{L^2(0,\ell _0)}=\frac{1}{2}\Vert \varepsilon u^\varepsilon _t(0,\cdot )\Vert ^2_{L^2(0,\ell _0)}$ tends to zero. The specific choice made in (4.1) simplifies the forthcoming computations; however, the same qualitative behaviour can be observed even without perturbations.

### Analysis of Dynamic Solutions

We now study the solutions $(u^\varepsilon ,\ell ^\varepsilon )$ to the coupled problem (2.1) and (2.14). Using (2.4) and (2.6) we find the following expression for $f^\varepsilon$ in $[-2\varepsilon ,2\varepsilon ]$,

4.2 $$\begin{aligned} f^\varepsilon (t) = {\left\{ \begin{array}{ll} f_1^\varepsilon (t):=\frac{\varepsilon +\sqrt{1+\varepsilon ^2}}{2}t+\varepsilon ^2\left\lfloor \frac{1}{\varepsilon }\right\rfloor , &{}\; a^\varepsilon _0\le t\le a^\varepsilon _1, \\ f_2^\varepsilon (t):=\frac{\varepsilon +\sqrt{1+\varepsilon ^2}}{2}t-\varepsilon \left\lfloor \frac{1}{\varepsilon }\right\rfloor t, &{}\; a^\varepsilon _1\le t\le a^\varepsilon _2, \\ f_3^\varepsilon (t):=\frac{\varepsilon +\sqrt{1+\varepsilon ^2}}{2}t-\varepsilon ^2\left\lfloor \frac{1}{\varepsilon }\right\rfloor , &{} \;a^\varepsilon _2\le t\le a^\varepsilon _3, \end{array}\right. } \end{aligned}$$

where $$a^\varepsilon _0:=-\varepsilon \ell _0=-2\varepsilon $$, $$a^\varepsilon _1:=-\varepsilon $$, $$a^\varepsilon _2:=\varepsilon $$, and $$a^\varepsilon _3=2\varepsilon $$.

Notice that $\dot{f}^\varepsilon$ is constant in every interval $(a^\varepsilon _{i-1},a^\varepsilon _{i})$, for $i=1,2,3$.

By (2.14) we have

4.3 $$\begin{aligned} \dot{\ell }^\varepsilon (t) = {\left\{ \begin{array}{ll} \dot{\ell }_1^\varepsilon :=\frac{1}{\varepsilon }\frac{2\left( \dot{f}_1^\varepsilon \right) ^2-\kappa }{2\left( \dot{f}_1^\varepsilon \right) ^2+\kappa }\vee 0=\frac{1}{\sqrt{1+\varepsilon ^2}}, &{} \;b^\varepsilon _0< t< b^\varepsilon _1, \\ \dot{\ell }_2^\varepsilon :=\frac{1}{\varepsilon }\frac{2\left( \dot{f}_2^\varepsilon \right) ^2-\kappa }{2\left( \dot{f}_2^\varepsilon \right) ^2+\kappa }\vee 0=0, &{}\; b^\varepsilon _1< t< b^\varepsilon _2, \\ \dot{\ell }_3^\varepsilon :=\frac{1}{\varepsilon }\frac{2\left( \dot{f}_3^\varepsilon \right) ^2-\kappa }{2\left( \dot{f}_3^\varepsilon \right) ^2+\kappa }\vee 0= \frac{1}{\sqrt{1+\varepsilon ^2}}&{} \;b^\varepsilon _2< t < b^\varepsilon _3, \end{array}\right. } \end{aligned}$$

where $$b^\varepsilon _0:=0$$ and

4.4 $$\begin{aligned} b^\varepsilon _i := b^\varepsilon _{i-1} + \frac{1}{1-\varepsilon \dot{\ell }^\varepsilon _i}\left( a^\varepsilon _i-a^\varepsilon _{i-1}\right) . \end{aligned}$$

Since $\dot{f}^\varepsilon$ is constant in $(a^\varepsilon _{i-1},a^\varepsilon _{i})$ for $i=1,2,3$, also $\dot{\ell }^\varepsilon$ is constant in the intervals $(b^\varepsilon _{i-1},b^\varepsilon _{i})$. We obtain $b^\varepsilon _1=\frac{1}{1-\varepsilon \dot{\ell }^\varepsilon _i} \varepsilon$, $b^\varepsilon _2=b^\varepsilon _1+2\varepsilon$, and $b^\varepsilon _3=b^\varepsilon _2 + \frac{1}{1-\varepsilon \dot{\ell }^\varepsilon _i} \varepsilon$.

We remark that in (4.3) $\dot{\ell }^\varepsilon _2=0$ because of (2.11). Indeed, for every $\varepsilon >0$ we have $2(\dot{f}_2^\varepsilon )^2 \le \kappa$ since

4.5 $$\begin{aligned} \left| \varepsilon + \sqrt{1+\varepsilon ^2} - 2\varepsilon \left\lfloor \frac{1}{\varepsilon }\right\rfloor \right| \le 1. \end{aligned}$$

Then (2.11b) is satisfied as a strict inequality, by (2.13), and therefore, (2.11c) implies that the debonding speed in this second interval is zero.

We now determine $f^\varepsilon$ for $t\ge a^\varepsilon _3$ and $\ell ^\varepsilon$ for $t\ge b^\varepsilon _3$ by using (2.5) and (2.14) recursively, cf. e.g. the proof of Proposition 3.1 for a similar construction. Because of (4.2) and (4.3), we can immediately see that $\dot{f}^\varepsilon$ and $\dot{\ell }^\varepsilon$ are piecewise constant. More precisely, $\dot{f}^\varepsilon (t)=\dot{f}^\varepsilon _i$ in each interval $(a^\varepsilon _{i-1},a^\varepsilon _i)$, $i>0$, while $\dot{\ell }^\varepsilon (t)=\dot{\ell }^\varepsilon _i$ in each interval $(b^\varepsilon _{i-1},b^\varepsilon _i)$, $i>0$, where

4.6 $$\begin{aligned} a^\varepsilon _{i+3}=a^\varepsilon _{i+2} + \frac{1+\varepsilon \dot{\ell }^\varepsilon _{i}}{1-\varepsilon \dot{\ell }^\varepsilon _{i}}\left( a^\varepsilon _{i}-a^\varepsilon _{i-1}\right) \end{aligned}$$

and $b^\varepsilon _i$ is given by (4.4). Notice that we have used (2.5) to obtain (4.6). Using (3.6) and recalling that $\dot{w}^\varepsilon = 1$, we get

4.7 $$\begin{aligned} \dot{f}^\varepsilon _{i+3} = \varepsilon + \frac{1-\varepsilon \dot{\ell }^\varepsilon _i}{1+\varepsilon \dot{\ell }^\varepsilon _i} \dot{f}^\varepsilon _i. \end{aligned}$$

Whenever $\dot{\ell }^\varepsilon _i=0$, then $\dot{f}^\varepsilon _{i+3} = \varepsilon + \dot{f}^\varepsilon _i$. On the other hand, when $\dot{\ell }^\varepsilon _i >0$, we can plug (2.14) in (4.7), which gives

4.8 $$\begin{aligned} \dot{f}^\varepsilon _{i+3} = \varepsilon + \frac{1-\frac{2\left( \dot{f}^\varepsilon _i\right) ^2 -\kappa }{2\left( \dot{f}^\varepsilon _i\right) ^2 +\kappa }}{1+\frac{2\left( \dot{f}^\varepsilon _i\right) ^2 -\kappa }{2\left( \dot{f}^\varepsilon _i\right) ^2 +\kappa }} \dot{f}^\varepsilon _i= \varepsilon + \frac{\kappa }{2 \dot{f}^\varepsilon _i}. \end{aligned}$$

This suggests us to study the map $h :x \mapsto \varepsilon + \frac{\kappa }{2x}$, which has a fixed point for $\bar{x} = \frac{\varepsilon + \sqrt{2\kappa +\varepsilon ^2}}{2}= \frac{\varepsilon + \sqrt{1 +\varepsilon ^2}}{2}$. Notice that $\bar{x} = \dot{f}^\varepsilon _1$. This implies that $\dot{f}^\varepsilon _{3i}=\dot{f}^\varepsilon _{3i+1}=\dot{f}^\varepsilon _1$ and $\dot{\ell }^\varepsilon _{3i}=\dot{\ell }^\varepsilon _{3i+1}=\dot{\ell }^\varepsilon _1$ for $i\ge 1$. In fact, the choice of the initial datum $u_0^\varepsilon$ as in (4.1a) has been made in order to satisfy these conditions and to simplify such formulas.

We still have to determine $\dot{f}^\varepsilon _{3i+2}$ and $\dot{\ell }^\varepsilon _{3i+2}$ for $i\ge 1$. To this end, we start by showing the explicit expression of $\ell ^\varepsilon$ in the interval $(b^\varepsilon _3, b^\varepsilon _6)$. By (4.3) and (4.6), we find

$$\begin{aligned} a^\varepsilon _4= a^\varepsilon _3 + c_\varepsilon \varepsilon , \quad a^\varepsilon _5=a^\varepsilon _4+2\varepsilon , \quad a^\varepsilon _6=a^\varepsilon _5+c_\varepsilon \varepsilon , \end{aligned}$$

where

$$\begin{aligned} c_\varepsilon :=\frac{1+\varepsilon \dot{\ell }^\varepsilon _1}{1-\varepsilon \dot{\ell }^\varepsilon _1}=1+\frac{2\varepsilon }{\sqrt{1+\varepsilon ^2}-\varepsilon }. \end{aligned}$$

We have already observed that $\dot{f}^\varepsilon _6=\dot{f}^\varepsilon _4 = \dot{f}^\varepsilon _1$. Moreover, by (4.7) and since $\dot{\ell }^\varepsilon _2=0$, we find $\dot{f}_5^\varepsilon = \dot{f}_2^\varepsilon + \varepsilon$. It easily follows that

$$\begin{aligned} \dot{\ell }^\varepsilon (t) = {\left\{ \begin{array}{ll} \dot{\ell }_4^\varepsilon =\dot{\ell }^\varepsilon _1=\frac{1}{\sqrt{1+\varepsilon ^2}}, &{}\; b^\varepsilon _3< t< b^\varepsilon _4, \\ \dot{\ell }_5^\varepsilon =\dot{\ell }^\varepsilon _2=0, &{} \;b^\varepsilon _4< t< b^\varepsilon _5, \\ \dot{\ell }_6^\varepsilon =\dot{\ell }^\varepsilon _1= \frac{1}{\sqrt{1+\varepsilon ^2}}&{}\; b^\varepsilon _5< t < b^\varepsilon _6. \end{array}\right. } \end{aligned}$$

Notice that $\dot{\ell }_5^\varepsilon =0$ holds for $\varepsilon$ small enough, since

4.9 $$\begin{aligned} \left| 3\varepsilon + \sqrt{1+\varepsilon ^2} - 2\varepsilon \left\lfloor \frac{1}{\varepsilon }\right\rfloor \right| \le 1 \end{aligned}$$

and therefore $2(\dot{f}_5^\varepsilon )^2 = 2(\dot{f}_2^\varepsilon +\varepsilon )^2 \le \kappa$, cf. (4.5).

We can iteratively repeat this argument as long as the following condition, analog of (4.5) and (4.9), is satisfied:

4.10 $$\begin{aligned} \left| (2i+1)\varepsilon + \sqrt{1+\varepsilon ^2} - 2\varepsilon \left\lfloor \frac{1}{\varepsilon }\right\rfloor \right| \le 1. \end{aligned}$$

Let

$$\begin{aligned} n^\varepsilon = \min \left\{ n \in \mathbb {N}: \left| (2n+1)\varepsilon + \sqrt{1+\varepsilon ^2} - 2\varepsilon \left\lfloor \frac{1}{\varepsilon }\right\rfloor \right| > 1\right\} . \end{aligned}$$

Notice that (2.11) implies that $2(\dot{f}_{3i+2}^\varepsilon )^2 = 2(\dot{f}_2^\varepsilon +i\varepsilon )^2 \le \kappa$ and $\dot{\ell }^\varepsilon _{3i-1}=0$ for every $i<n^\varepsilon$. Condition (4.10) is a threshold condition that fails after $n^\varepsilon$ iterations of this process. Direct computations show that $n^\varepsilon = \lfloor \frac{1}{\varepsilon } \rfloor$. (In fact, the choice of the initial datum $u_0^\varepsilon$ has been made in order to obtain this equality.) In conclusion, for $\varepsilon$ sufficiently small and $1 \le i < n^\varepsilon$, we have

4.11 $$\begin{aligned} \dot{f}^\varepsilon (t)= {\left\{ \begin{array}{ll} \dot{f}^\varepsilon _{3i+1} = \dot{f}^\varepsilon _1, &{}\; a^\varepsilon _{3i}< t< a^\varepsilon _{3i+1}, \\ \dot{f}^\varepsilon _{3i+2} = \dot{f}^\varepsilon _2 + i\varepsilon , &{}\; a^\varepsilon _{3i+1}< t< a^\varepsilon _{3i+2}, \\ \dot{f}^\varepsilon _{3i+3} = \dot{f}^\varepsilon _1, &{}\; a^\varepsilon _{3i+2}< t < a^\varepsilon _{3i+3}, \end{array}\right. } \end{aligned}$$

and

4.12 $$\begin{aligned} \dot{\ell }^\varepsilon (t)= {\left\{ \begin{array}{ll} \dot{\ell }_{3i+1}^\varepsilon =\dot{\ell }^\varepsilon _1, &{}\; b^\varepsilon _{3i}< t< b^\varepsilon _{3i+1}, \\ \dot{\ell }_{3i+2}^\varepsilon =0, &{}\; b^\varepsilon _{3i+1}< t< b^\varepsilon _{3i+2}, \\ \dot{\ell }_{3i+3}^\varepsilon =\dot{\ell }^\varepsilon _1, &{} \;b^\varepsilon _{3i+2}< t < b^\varepsilon _{3i+3}, \\ \end{array}\right. } \end{aligned}$$

where

4.13 $$\begin{aligned} {\left\{ \begin{array}{ll} a^\varepsilon _{3i+1} =a^\varepsilon _{3i} + c_\varepsilon \left( a^\varepsilon _{3i-2}-a^\varepsilon _{3i-3}\right) =2\varepsilon (i-1)+2\varepsilon \frac{1-c_\varepsilon ^i}{1-c_\varepsilon } + c_\varepsilon \varepsilon ,\\ a^\varepsilon _{3i+2} =a^\varepsilon _{3i+1} + 2\varepsilon =2\varepsilon i+2\varepsilon \frac{1-c_\varepsilon ^i}{1-c_\varepsilon } + c_\varepsilon \varepsilon ,\\ a^\varepsilon _{3i+3} =a^\varepsilon _{3i+2} +c_\varepsilon \left( a^\varepsilon _{3i}-a^\varepsilon _{3i-1}\right) =2\varepsilon i+2\varepsilon \frac{1-c_\varepsilon ^{i+1}}{1-c_\varepsilon },\\ b^\varepsilon _{3i-2} =b^\varepsilon _{3i-3} + \frac{1}{1-\varepsilon \dot{\ell }_1^\varepsilon } \left( a^\varepsilon _{3i-2}-a^\varepsilon _{3i-3}\right) = 2\varepsilon i + \frac{c_\varepsilon ^i-1}{\dot{\ell }^\varepsilon _1} + 2\varepsilon \frac{1}{1-\varepsilon \dot{\ell }_1^\varepsilon }c_\varepsilon ^i,\\ b^\varepsilon _{3i-1} =b^\varepsilon _{3i-2} + 2\varepsilon =2\varepsilon (i+1) + \frac{c_\varepsilon ^i-1}{\dot{\ell }^\varepsilon _1} + 2\varepsilon \frac{1}{1-\varepsilon \dot{\ell }_1^\varepsilon }c_\varepsilon ^i,\\ b^\varepsilon _{3i} =b^\varepsilon _{3i-1} + \frac{1}{1-\varepsilon \dot{\ell }_1^\varepsilon } \left( a^\varepsilon _{3i}-a^\varepsilon _{3i-1}\right) = 2\varepsilon i + \frac{c_\varepsilon ^{i+1}-1}{\dot{\ell }^\varepsilon _1}. \end{array}\right. } \end{aligned}$$

**Fig. 5:**
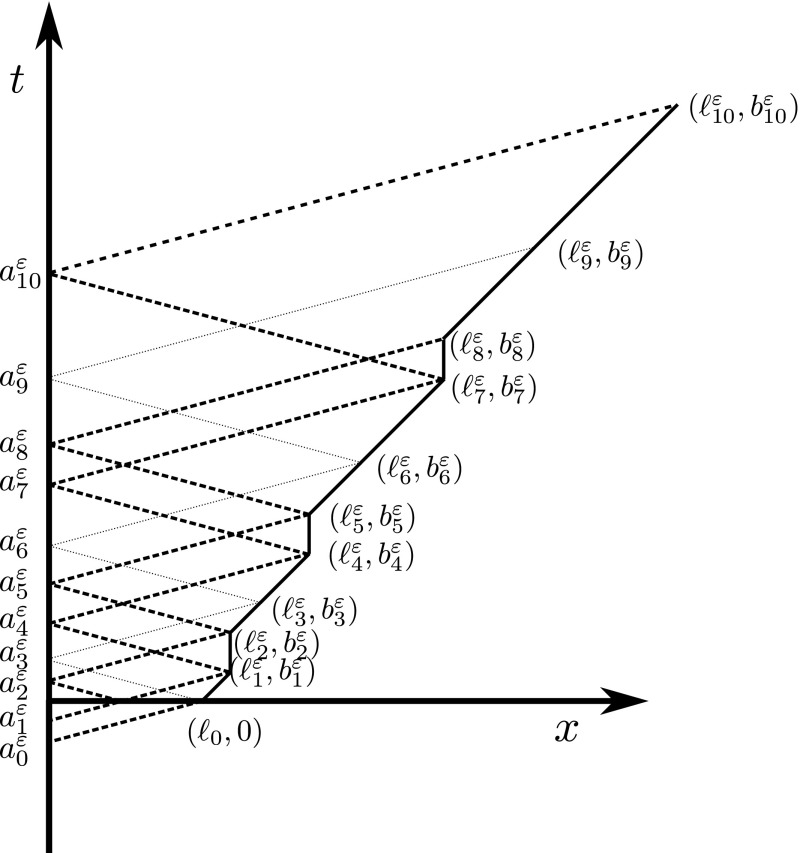
Evolution of $\ell ^\varepsilon$ according to a “stop and go” process. Since $\dot{f}^\varepsilon$ is constant in each of the intervals $(a_i^\varepsilon , a_{i+1}^\varepsilon )$, by (2.7) the displacement’s derivatives are constant in each of the sectors bounded by *dashed lines*. It turns out that *thick dashed lines* are in fact shock waves, while *thin dashed lines* are *not* discontinuity lines for the derivatives (cf. Remark 4.1)

This means that there is a first phase, corresponding to the time interval $[0,b^\varepsilon _{3n^\varepsilon }]$, where the material debonds according to a “stop and go” process and the speed oscillates between 0 and $\dot{\ell }_1^\varepsilon$ (see Fig. 5).

Let us now consider the evolution for times larger than $b^\varepsilon _{3n^\varepsilon }$. Arguing as above, we obtain

$$\begin{aligned} \dot{f}_{3n^\varepsilon +2}^\varepsilon = \frac{\varepsilon + \sqrt{1+\varepsilon ^2}}{2} = \dot{f}_1^\varepsilon \quad \text {and} \quad \dot{\ell }_{3n^\varepsilon +2}^\varepsilon = \dot{\ell }^\varepsilon _1 . \end{aligned}$$

We employ (4.8) and recall that the map $h :x \mapsto \varepsilon + \frac{\kappa }{2x}$ has a fixed point at $\bar{x}=\dot{f}_1^\varepsilon$. Therefore, for every $i \ge n^\varepsilon$,

$$\begin{aligned} {\left\{ \begin{array}{ll} \dot{f}_{3i+1}^\varepsilon = \dot{f}_{3i+2}^\varepsilon = \dot{f}_{3i+3}^\varepsilon = \dot{f}_1^\varepsilon , \\ \dot{\ell }_{3i+1}^\varepsilon = \dot{\ell }_{3i+2}^\varepsilon = \dot{\ell }_{3i+3}^\varepsilon = \dot{\ell }^\varepsilon _1. \end{array}\right. } \end{aligned}$$

This shows that in this second phase the debonding proceeds at constant speed $\dot{\ell }_1^\varepsilon$ for every time.

#### Remark 4.1

By (2.7), (4.2), and (4.11), the displacement’s derivatives are piecewise constant; in the (*t*, *x*) plane, their discontinuities lie on some shock waves originating from $(0,\ell _0/2)$ (where the initial datum has a kink), travelling backword and forward in the debonded film, and reflecting at boundaries; they are represented by thick dashed lines in Fig. 5. Notice that the lines originating from $(0,\ell _0)$, employed in the construction above and marked by thin dashed lines in Fig. 5, are *not* discontinuity lines, since $\dot{f}^\varepsilon _{3i} = \dot{f}^\varepsilon _{3i+1}$ for every $i\ge 1$. This is actually a consequence of the compatibility among $u^\varepsilon _0$, $u_1^\varepsilon$, and $\dot{\ell }^\varepsilon$ at $(0,\ell _0)$, namely $\dot{u}^\varepsilon _0(\ell _0)\dot{\ell }^\varepsilon (0)+u_1^\varepsilon (\ell _0)=0$. We refer to Dal Maso et al. (2016, Remark 1.12) for more details on the regularity of the solutions.

### Limit for Vanishing Inertia

We now study the limit $\ell$ of the evolutions $\ell ^\varepsilon$ as $\varepsilon \rightarrow 0$. Notice that the initial conditions are not at equilibrium; in particular the initial position $u_0(x)$ is not of the form $\big [-\frac{w(0)}{\ell _0}x+w(0)\big ] \vee 0$. Because of (3.11), there must be a time discontinuity at $t=0$, i.e. the limit displacement *u* jumps to an equilibrium configuration. Nonetheless, we will show that $\ell$ is continuous even at $t=0$. In order to determine $\ell$, the main point is to study the limit evolution of the debonding during the first phase characterised by the “stop and go” process illustrated above. Afterwards, during the second phase, the evolution of the debonding will proceed at constant speed, given by $\lim _{\varepsilon \rightarrow 0}\dot{\ell }^\varepsilon _1=1$.

We first compute the instant at which the first phase ends. By (4.13), we have

$$\begin{aligned} b^\varepsilon _{3n^\varepsilon } = 2\varepsilon \left\lfloor \frac{1}{\varepsilon } \right\rfloor + \frac{c_\varepsilon ^{\lfloor \frac{1}{\varepsilon } \rfloor +1}-1}{\dot{\ell }_1^\varepsilon } \;\mathop {\longrightarrow }\limits ^{\varepsilon \rightarrow 0} \; e^2+1 . \end{aligned}$$

On the other hand, at time $b^\varepsilon _{3n^\varepsilon }$ the position of $\ell ^\varepsilon$ is given by

4.14 $$\begin{aligned} \ell ^\varepsilon \left( b^\varepsilon _{3n^\varepsilon }\right) = \ell _0 + \left( b^\varepsilon _{3n^\varepsilon } - 2\varepsilon \left\lfloor \frac{1}{\varepsilon } \right\rfloor \right) \dot{\ell }_1^\varepsilon = \ell _0 + c_\varepsilon ^{\left\lfloor \frac{1}{\varepsilon } \right\rfloor +1}-1 \; \mathop {\longrightarrow }\limits ^{\varepsilon \rightarrow 0} \; e^2+1 . \end{aligned}$$

Indeed, in $[0,b^\varepsilon _{3n^\varepsilon }]$ the speed $\dot{\ell }^\varepsilon$ is either zero or $\dot{\ell }^\varepsilon _1$, and the total length of the intervals where $\dot{\ell }^\varepsilon =0$ is $2\varepsilon \lfloor \frac{1}{\varepsilon } \rfloor$.

Therefore, for $t\ge e^2{+}1$ we have $\ell (t)=t$. In the time interval $[e^2{+}1,+\infty )$, corresponding to the second phase, the quasistatic limit $\ell$ is a rate-independent evolution in the sense of Definition 3.6, see also Remark 3.8.

We now explicitly find the law of the evolution of $\ell$ in the first phase. Rather than seeking an expression for $t\mapsto \ell (t)$, it is more convenient to determine the inverse map $\ell \mapsto t(\ell )$, cf. Lazzaroni et al. (2012) for a similar computation in another example. We consider the map

4.15 $$\begin{aligned} i\mapsto \ell ^\varepsilon (b^\varepsilon _{3i}) = \ell _0 + c_\varepsilon ^{i+1}-1, \end{aligned}$$

where $1 \le i < n^\varepsilon$. Notice that the last equality follows as in (4.14). We now take the inverse of (4.15) and define

$$\begin{aligned} i^\varepsilon (\ell ) := \left\lfloor \frac{\log \left( \frac{1+\ell -\ell _0}{c_\varepsilon }\right) }{\log c_\varepsilon } \right\rfloor . \end{aligned}$$

Since $c_\varepsilon ^\frac{1}{\varepsilon } \rightarrow e^2$ as $\varepsilon \rightarrow 0$, then we have

4.16 $$\begin{aligned} \varepsilon i^\varepsilon (\ell ) \; \mathop {\longrightarrow }\limits ^{\varepsilon \rightarrow 0} \; \frac{\log \left( \ell -1\right) }{2} . \end{aligned}$$

**Fig. 6:**
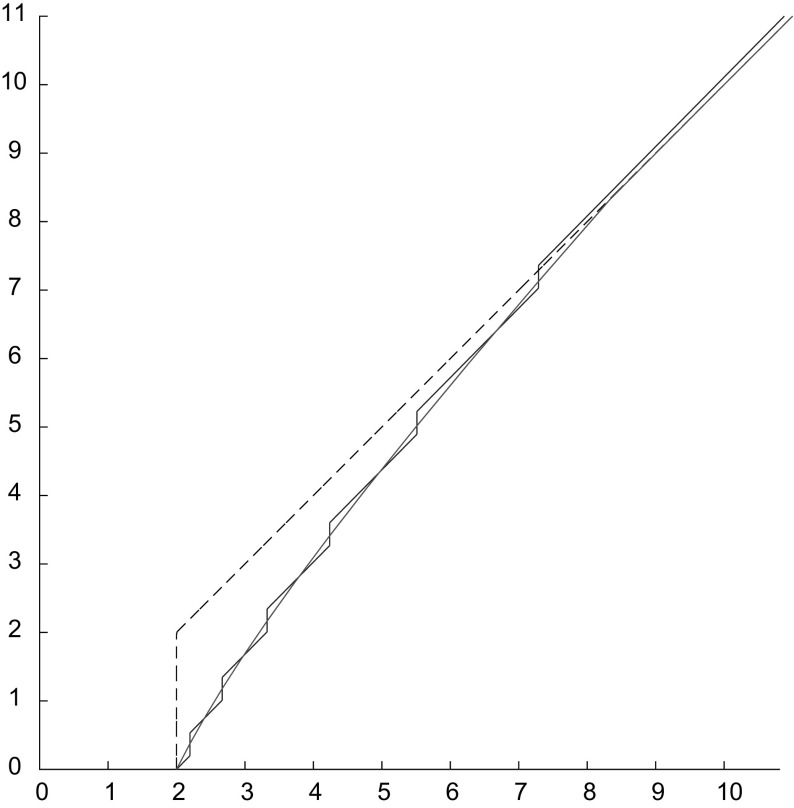
Dynamic solution for $\varepsilon =0.1$ (*zig-zag*), asymptotic limit (*continuous line*), and a rate-independent evolution (*dashed line*)

Therefore,

4.17 $$\begin{aligned} t(\ell )= & {} \lim _{\varepsilon \rightarrow 0} b^\varepsilon _{i^\varepsilon (\ell )} =\lim _{\varepsilon \rightarrow 0}\left( 2\varepsilon i^\varepsilon (\ell ) + \frac{c_\varepsilon ^{i^\varepsilon (\ell )+1}-1}{\dot{\ell }_1^\varepsilon }\right) \nonumber \\= & {} \log (\ell -1) + \ell -2, \quad \ell \in (\ell _0,e^2{+}1), \end{aligned}$$

denotes the trajectory followed by the debonding during the first phase (see Fig. 6). Notice that $t(\ell )$ is the sum of a strictly concave and an affine function, thus $\ell (t)$ is strictly convex in the first phase. It is interesting that the first phase features a strictly positive debonding acceleration.

We can give first- and second-order laws characterising the first phase. By (4.17) we obtain

$$\begin{aligned} \dot{t}(\ell )= \frac{\ell }{\ell -1} \quad \text {for } \ell \in (2,e^2{+}1), \end{aligned}$$

hence

$$\begin{aligned} \dot{\ell }(t)=\frac{\ell (t)-1}{\ell (t)} \quad \text {for } t\in (0,e^2{+}1) \end{aligned}$$

and

$$\begin{aligned} \ddot{\ell }(t)=\frac{\ell (t)-1}{\ell (t)^3} \quad \text {for } t\in (0,e^2{+}1). \end{aligned}$$

As already observed, we have $\dot{\ell },\ddot{\ell }>0$ in the first phase. Both $\dot{\ell }$ and $\ddot{\ell }$ are discontinuous at $t=e^2{+}1$.

Notice that during the first phase the quasistatic limit $\ell$ does not satisfy (3.20c), thus it does not comply with the notion of rate-independent evolution given in Definition 3.6. Indeed, since the local toughness is constant, Remark 3.8 implies that a rate-independent evolution must be piecewise affine (with possible jumps); in contrast, (4.17) is not the equation of a line. This result is similar to the one obtained in Lazzaroni et al. (2012) with a discontinuous local toughness: here we showed that a singular behaviour can be observed even if the local toughness is constant.

#### Remark 4.2

We recall that the initial displacement $u_0^\varepsilon$ chosen in (4.1a) has a kink at $\frac{\ell _0}{2}=1$. In this section, we showed that the interaction between the two slopes generates the “stop and go” process, which gives as a result the convergence to an evolution that does not satisfy Definition 3.6. However, this singular behaviour can be obtained even for a smooth initial datum. Indeed, let us consider a regularisation of $u_0^\varepsilon$, coinciding with the original profile outside of $(1-\frac{\delta }{2},1+\frac{\delta }{2})$, where $\delta \in (0,1)$ is fixed. As a consequence of this choice, the function $\dot{\ell }^\varepsilon$ differs from (4.12) only in a portion of the order $\varepsilon \delta$ of each interval $(b_i^\varepsilon ,b_{i+1}^\varepsilon )$. The resulting evolution of the debonding front $\ell ^\varepsilon$ is smooth. However, in the limit we observe the same qualitative behaviour described above, due to the interaction of the different slopes of the initial datum. This shows that the singular behaviour is not due to the choice of a initial datum with a kink.

### Analysis of the Kinetic Energy

The striking behaviour observed in the previous example can be explained by computing the oscillations of the kinetic energy

4.18 $$\begin{aligned} K^\varepsilon (t):=\frac{\varepsilon ^2}{2} \int _0^{\ell ^\varepsilon (t)} u_t^\varepsilon (t,x)^2 \mathrm {d}x . \end{aligned}$$

We recall that the displacement’s derivatives are piecewise constant, with discontinuity lines given by shock waves originating at $\ell _0/2$ (where the initial datum has a kink) and travelling backward and forward in the debonded film (cf. Remark 4.1).

**Fig. 7:**
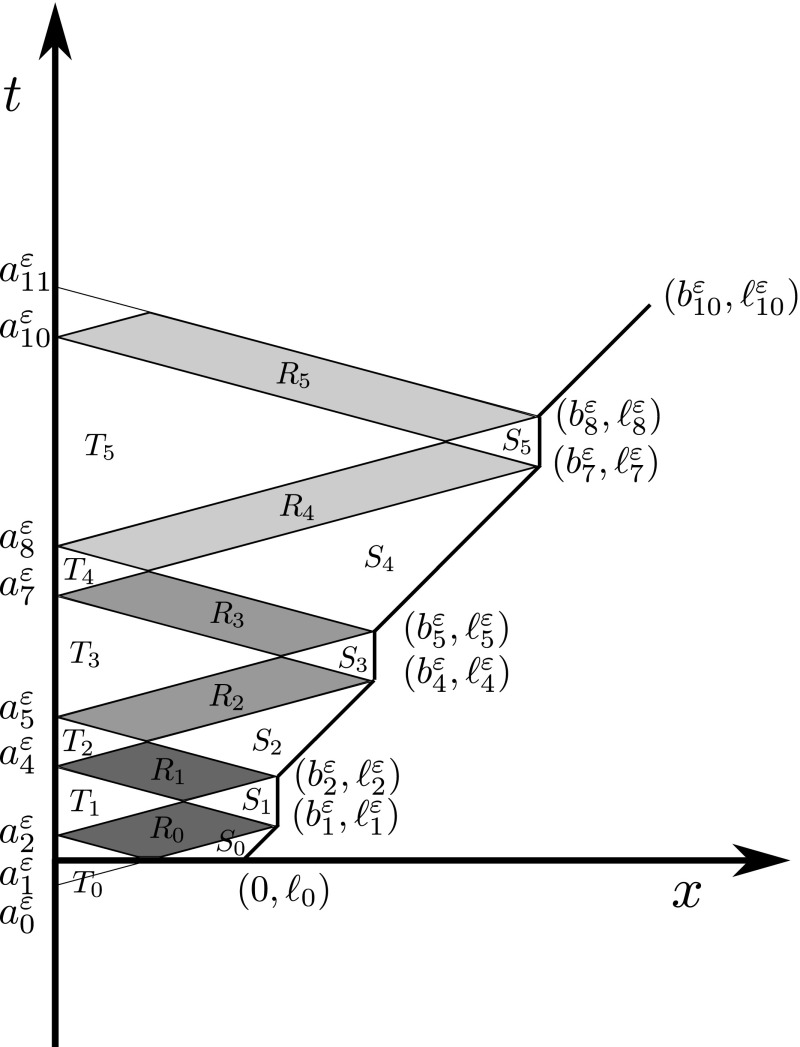
The sectors composing $\Omega ^\varepsilon$ give different contributions to the kinetic energy $K^\varepsilon$. The *darker* the shade of *grey*, the larger is $u^\varepsilon _t(t,x)^2$ in that region. White sectors give a negligible contribution

Let us introduce some notation. The sectors determined by shock waves (Fig. 7) are divided into three families: $T_i$ denotes a triangular sector adjacent to the time axis (i.e. the vertical axis in figure), $S_i$ a triangular sector adjacent to the graph of $\ell ^\varepsilon$, and $R_i$ a rhomboidal sector; $T_0$ contains the segment $\{0\}\times [a_1^\varepsilon ,a_2^\varepsilon ]$, $S_0$ contains the segment $[(0,\ell _0),(b_1^\varepsilon ,\ell _1^\varepsilon )]$, and $R_0$ is adjacent to $T_0$ and $S_0$; the families are indexed increasingly in the direction of the time axis.

It is easy to see that the boundary conditions imply that $u^\varepsilon _t=1$ in the sectors $T_i$ and $u^\varepsilon _t=0$ in the sectors $S_i$ with *i* odd, i.e. those triangles corresponding to a stop phase of the debonding front. Moreover, by (2.7a), (4.2), and (4.11) we obtain that $u^\varepsilon _t=1$ in the sectors $S_i$ with *i* even. In all triangular sectors we thus have $u_t^\varepsilon (t,x)$ of order at most one, so that their contribution to the kinetic energy (4.18) is of order at most $\varepsilon ^2$. More precisely,

$$\begin{aligned} \frac{\varepsilon ^2}{2} \int _{(\{t\}\times [0,{\ell ^\varepsilon (t)}])\cap T_i} u_t^\varepsilon (t,x)^2 \mathrm {d}x \le C \varepsilon ^2 \quad \text {for every } t,i, \end{aligned}$$

for some $C>0$, and the same holds for $S_i$. In particular,

4.19 $$\begin{aligned} K^\varepsilon (t)=O(\varepsilon ^2) \quad \text {for } t=\frac{a^\varepsilon _{3i-1}+a^\varepsilon _{3i+1}}{2}\quad \text {and for } t=\frac{a^\varepsilon _{3i+1}+a^\varepsilon _{3i+2}}{2} \end{aligned}$$

for every $$i\ge 1$$.

We now show that the remaining rhomboids $R_i$ give a relevant contribution to the kinetic energy. By (2.7a), (4.2), and (4.11) we obtain for every *i*

$$\begin{aligned} u_t^\varepsilon (t,x)&= 1 - \frac{1}{\varepsilon }\dot{f}^\varepsilon _1 +\frac{1}{\varepsilon }\left( \dot{f}^\varepsilon _1 -\varepsilon \left\lfloor \frac{1}{\varepsilon }\right\rfloor + i\varepsilon \right) =1 - \left\lfloor \frac{1}{\varepsilon }\right\rfloor +i \quad \text {in } R_{2i}, \\ u_t^\varepsilon (t,x)&=1 - \frac{1}{\varepsilon }\left( \dot{f}^\varepsilon _1 -\varepsilon \left\lfloor \frac{1}{\varepsilon }\right\rfloor + (i+1)\varepsilon \right) +\frac{1}{\varepsilon }\dot{f}^\varepsilon _1 = 1 + \left\lfloor \frac{1}{\varepsilon }\right\rfloor -(i+1) \quad \text {in } R_{2i+1}. \end{aligned}$$

To obtain the kinetic energy (4.18), we observe that the maximal *t*-section of each rhomboid has length $\ell _0=2$. Therefore,

4.20 $$\begin{aligned} K^\varepsilon (t) = \varepsilon ^2 \left( \left\lfloor \frac{1}{\varepsilon }\right\rfloor -i \right) ^2 + O(\varepsilon ) \quad \text {for } t\in \left[ a^\varepsilon _{3i+2}, b^\varepsilon _{3i+1}\right] \cup \left[ b^\varepsilon _{3i+2}, a^\varepsilon _{3i+4}\right] . \end{aligned}$$

This gives the maximal asymptotic amount of kinetic energy; we do not detail the computation of the kinetic energy in other intervals. We recall that by (4.19) the minimal asymptotic amount is zero, so the energy is oscillating.

Moreover, since (4.20) holds for $i = 0, \ldots , n^\varepsilon$ and since $n^\varepsilon = \lfloor \frac{1}{\varepsilon } \rfloor$, we observe that the maximal oscillations of the kinetic energy decrease as time increases, until the kinetic energy is close to zero for $i=n^\varepsilon$, i.e. when the non-quasistatic phase finishes and the second phase starts. In fact, since in the second phase $\dot{f}^\varepsilon (t) = \dot{f}^\varepsilon _1$ for a.e. *t*, then $u^\varepsilon _t$ is constantly equal to one, so the kinetic energy is negligible by (4.18). We can also give an asymptotic expression for the maximal (resp., minimal) oscillations by plugging (4.16) in (4.20) [resp., by (4.19)]:

4.21 $$\begin{aligned} \mathop {\Gamma \hbox {-}\lim }_{\varepsilon \rightarrow 0}{(-K^\varepsilon )}(t)=-\left( 1- \frac{\log (\ell (t)-1)}{2} \right) ^{2}, \quad \mathop {\Gamma \hbox {-}\lim }_{\varepsilon \rightarrow 0}{(K^\varepsilon )}(t)=0 . \end{aligned}$$

We refer to Braides (2006) for the notion of $\Gamma$-convergence. A similar phenomenon was observed in Lazzaroni et al. (2012) for a discontinuous toughness.

Summarising,

- the non-quasistatic phase, where Griffith’s quasistatic criterion fails in the limit, is characterised by the presence of a relevant kinetic energy (of order one as $\varepsilon \rightarrow 0$, at each fixed time);
- during such first phase, kinetic energy oscillates and is exchanged with potential energy at a timescale of order $\varepsilon$;
- overall, the total mechanical energy decreases and is transferred into energy dissipated in the debonding growth;
- as time increases, the maximal oscillations of the kinetic energy decrease and approach zero as $t\rightarrow e^2+1$, i.e. all of the kinetic energy is converted into potential and dissipated energy;
- in the second (stable) phase, for $t\ge e^2+1$, the kinetic energy is of order $\varepsilon ^2$ and does not influence the limit behaviour of the debonding evolution as $\varepsilon \rightarrow 0$.

## Conclusion

In this paper we have studied a dynamic peeling test and its limit for slow loading (or, equivalently, for vanishing inertia). We have proved that the limit displacement is at equilibrium (i.e. it is affine, see Theorem 3.5), while the limit debonding evolution is non-decreasing and satisfies a stability condition, precisely the energy release rate is controlled by the local toughness (Theorem 3.11). In contrast, the activation condition (3.20c) in Griffith’s criterion is in general not satisfied in the limit as shown by the example in Sect. 4. In fact, in that case the quasistatic energy balance does not hold, because of the presence of a relevant amount of kinetic energy during a first phase [whose asymptotic limit is given by (4.17) and (4.21)].

Such phase features a strictly positive debonding acceleration. In fact, in fracture mechanics, it was observed that the equation of motion involves crack acceleration in finite size specimens, as that considered here (Marder 1991; Goldman et al. 2010). The emergence of crack inertia is due to waves generated by the crack that interact with the crack after bouncing on the boundary. A similar phenomenon is observed in our example for the peeling test. The role of debonding acceleration in the quasistatic limit will be matter of future investigation: this may lead to a better characterisation of the notion of solution found in the limit.

Our example highlights the relevance of dynamical effects in debonding propagation under quasistatic loading: the quasistatic approximation given by Griffith’s criterion is not appropriate in this case since the kinetic energy can not be neglected. A similar behaviour was observed in Lazzaroni et al. (2012), arising from toughness defects. Our example shows a new situation where convergence to a rate-independent solution fails, due to initial data out of equilibrium. The same phenomenon is observed if we choose as initial condition $u^\varepsilon _0(x):=u^\varepsilon ((a^\varepsilon _{3i+1}+a^\varepsilon _{3i+2})/2,x)$ (with the notation of the previous section), thus we obtain a non-quasistatic propagation even starting from an initial datum arbitrarily close to equilibrium.

Hence, our new example indicates that, in order to get convergence to Griffith’s criterion, one should essentially consider the trivial case of an initial datum at equilibrium. Indeed, also in finite-dimensional singularly perturbed second-order potential-type equations, convergence to equilibrium is enforced by taking initial conditions at equilibrium (Nardini 2017); however, choosing an initial datum at equilibrium is not needed if such equations include a viscosity term tending to zero as inertia vanishes (Agostiniani 2012). This suggests that, in the case of the peeling test, Griffith’s criterion may hold in the quasistatic limit if the dynamic equations are damped. We leave this question open for further research.
